# Noncovalent Metal–Water Polarization Governs Helmholtz Capacitance

**DOI:** 10.1021/jacs.6c11740

**Published:** 2026-10-24

**Authors:** Lang Li, Elias Diesen, Nicolas G. Hörmann, Karsten Reuter

**Affiliations:** Theory Department, 28259 Fritz-Haber-Institut der Max-Planck-Gesellschaft, Faradayweg 4-6, 14195 Berlin, Germany

## Abstract

Electrified metal–water interfaces govern charge storage and electrochemical reactivity, yet the microscopic origin of their metal-dependent Helmholtz capacitance has remained unresolved. Here, we use extensive density functional theory-based *ab initio* molecular dynamics simulations to reproduce and explain the long-established experimental capacitance trend at fcc(111) metal–water interfaces. We show that this trend is governed primarily by the electrode-side electronic response of the explicit metal–water interface, which controls the effective electron spillover length and, hence, the bias capacitance. This relation remains predictive for cross-geometry Pt/Au systems. Our findings clarify the microscopic origin of metal-dependent Helmholtz capacitance and suggest a physically grounded design principle for electrode materials in sustainable-energy technologies.

## Graphical abstract

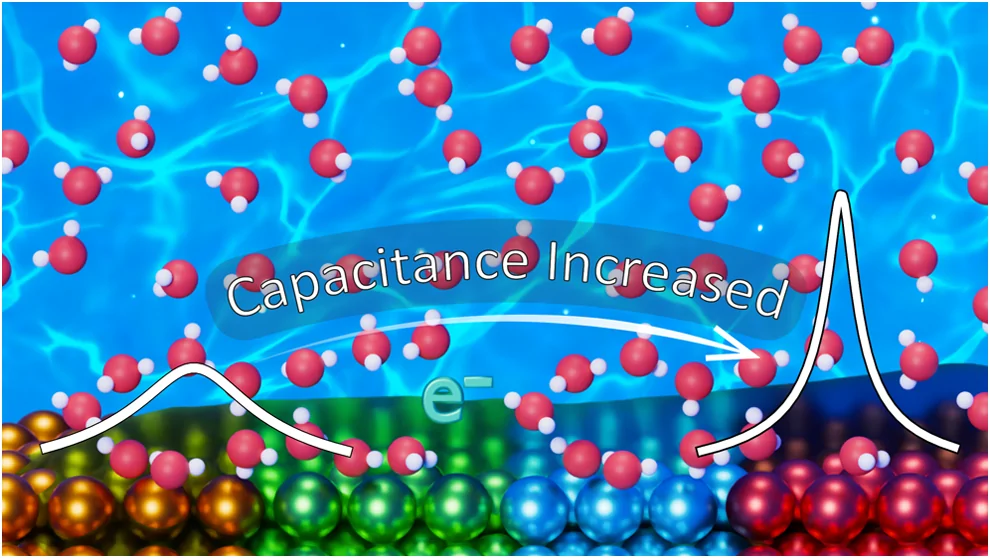

## Introduction

The electric double layer (EDL), formed at the interface between a charged surface and an electrolyte, governs charge storage, interfacial reactivity, and ionic organization across a wide range of systems, including energy storage devices, electrocatalytic interfaces, colloidal dispersions, sensors, and biointerfaces.^1–6^ Owing to this central role, the EDL has been studied extensively,^7–9^ with particular attention to its differential capacitance *C*, which quantifies the change in surface charge with electrode potential. *C* can straightforwardly be measured by cyclic voltammetry and electrochemical impedance spectroscopy.^10–19^ Its microscopic interpretation, however, remains nontrivial, especially when double-layer charging and surface redox processes coexist, blurring the distinction between double-layer capacitance and pseudocapacitance.^20^ In this context, well-defined single-crystal metal–water interfaces provide an important benchmark because their capacitance can be both measured experimentally and resolved by atomistic simulation.^10–19,21–29^

A long-standing goal has therefore been to establish a universal explanation for the observed double-layer capacitance. In the classical Gouy–Chapman–Stern picture, the total capacitance is described as a series combination of a diffuse-layer capacitance and a compact-layer, or Helmholtz, capacitance

1 $$\frac{1}{C}=\frac{1}{C_{H}}+\frac{1}{C_{\mathrm{GC}}}$$

This framework captures the dominant influence of the diffuse layer at low electrolyte concentrations, while predicting that at sufficiently high concentrations, the total capacitance should approach the Helmholtz limit.^30^ Experimentally, however, crystalline metal electrodes exhibit pronounced capacitance peaks near the potential of zero charge (PZC),^15,16,19,30,31^ and this Helmholtz layer-dominated response depends strongly on the metal identity even among closely related fcc(111) noble metals.^12–14,32^ Although the Gouy–Chapman–Stern framework rationalizes some idealized cases, such as Hg,^33^ it explains neither the pronounced PZC-centered peaks nor their systematic metal dependence at crystalline metal–water interfaces.

Since the 1960s, explanations of metal-dependent Helmholtz capacitance have largely emphasized one of three microscopic ingredients: the metal electronic response, the water response, or the metal–water interaction. Early metal-centered descriptions proposed that Helmholtz capacitance is linked to the electronic properties of the electrode.^34–40^ In particular, Trasatti argued that for simple *sp* metals near the PZC, the capacitance correlates with electronic density.^38^ However, such treatments do not explicitly resolve atomistic lattice effects or the response to interfacial water, limiting their applicability for transition and noble metals such as Pt, Cu, Ag, and Au. By contrast, water-focused models, ranging from orientational descriptions to multistate interfacial water pictures,^41,42^ emphasized solvent polarization but did not explicitly capture metal-dependent polarization of interfacial water, hydrogen bond-network effects, or their interplay with metal–water bonding. A third line of work emphasized the metal–water interaction itself, beginning with the distinction between chemisorbed and nonchemisorbed interfacial water introduced by Damaskin and Frumkin^43^ and later developed by Parsons.^44^ More recently, density functional theory-based *ab initio* molecular dynamics (DFT-AIMD) has enabled explicit atomistic descriptions of electrified metal–water interfaces. In particular, Cheng and co-workers showed that potential-dependent water chemisorption can account for characteristic capacitance peaks on Pt and Ag.^24,27^ Still, a unified microscopic explanation for the broader metal-dependent Helmholtz capacitance trend remains lacking.

Here, we address this problem using extensive DFT-AIMD simulations of the EDL at four fcc(111) metal surfaces, Pt, Cu, Ag, and Au. To test the transferability of the resulting picture (including atomistic lattice effects), we further consider two cross-geometry systems in which the lattice constants of Pt and Au are interchanged, henceforth denoted as Pt@Au and Au@Pt, respectively. Our calculations fully reproduce the long-established experimental Helmholtz capacitance trend^12–14,17–19,32^ and show that it can be understood within a unified description of the interfacial response. We find that this trend is governed primarily by the electrode-side electronic response generated at the explicit metal–water interface, through its control of the effective electron spillover length and the associated bias capacitance. By contrast, the interfacial water response shows no systematic metal dependence, instead fluctuating around an approximately constant value. The same framework also captures the capacitance variations of the cross-geometry systems, demonstrating its transferability. Together, these findings establish a coherent quantum-mechanical description of the metal–water EDL, clarify the microscopic origin of metal-dependent Helmholtz capacitance, and provide a physically grounded design principle for electrode materials.

## Results and Discussion

### Metal-Dependent Double-Layer Capacitance

Figure 1 shows the differential capacitance of electrified fcc(111) metal–water interfaces obtained from DFT-AIMD and compared with the experiment. Within experimental uncertainties, the peak capacitance follows the long-established trend, Pt(111) > Cu(111) ∼ Ag(111) > Au(111), which is fully reproduced by the simulations with peak values of *C*_max_ of approximately 100, 60, 50, and 40 μF cm^–2^, respectively. This establishes our DFT-AIMD framework as a reliable basis for resolving the microscopic origin of the metal dependence discussed below.

**Figure 1:**
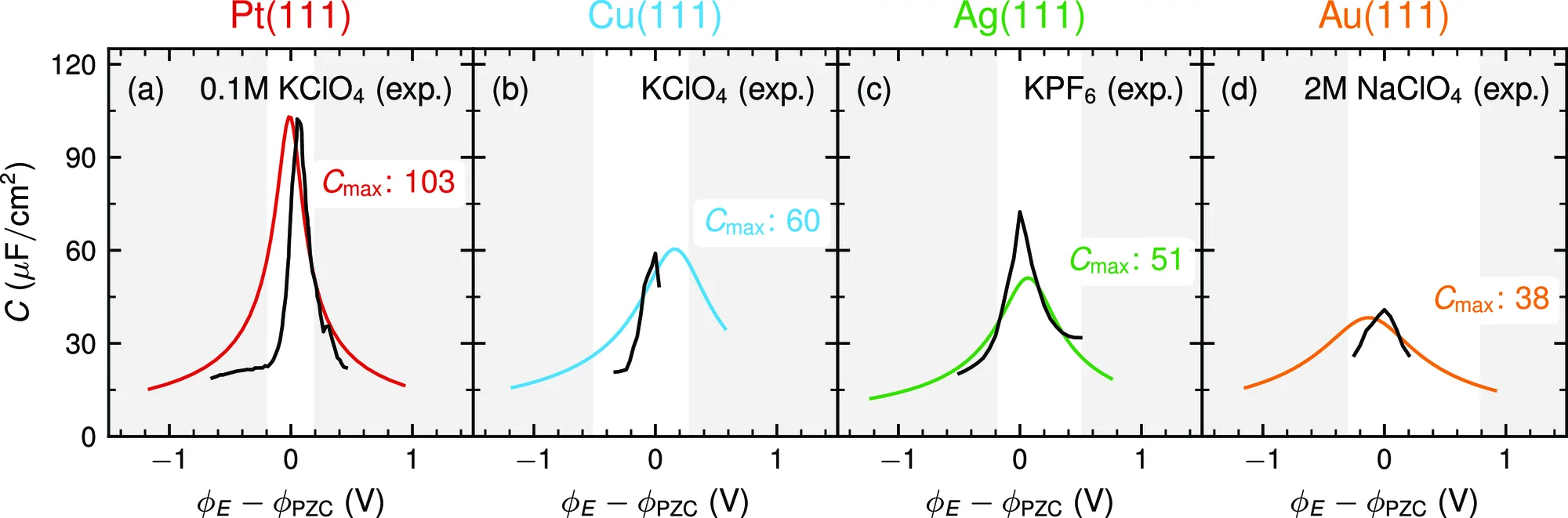
Differential capacitance of electrified metal/water interfaces. Experimental differential capacitance data were adapted with permission from the published electrochemical impedance spectroscopy measurements for Pt(111) in 0.1M KClO_4_,^16^ Cu(111) in aqueous KClO_4_,^17^ Ag(111) in aqueous KPF_6_,^18^ and Au(111) in 2 M NaClO_4_.^19^ Copyright 2001, 1985, and 1989 Elsevier for refs,^16–18^ respectively; Copyright 2025 by the American Physical Society for ref 19. See Section S2 of the SI for details. For Cu(111) and Ag(111), the experimental curves are shown after Parsons–Zobel analysis to extrapolate the Helmholtz capacitance.^30^ DFT-AIMD results are shown as colored curves (Pt, red; Cu, blue; Ag, green; Au, orange). Gray-shaded regions indicate charge ranges that may contain artifacts from electronic instability; see Figures S9 and S16 and Section S3 of the SI and our previous work.^21^ Labels indicate the peak capacitance, *C*_max_, extracted from the DFT-AIMD results.

To obtain these results, we employ the DFT-PCH (partially charged hydrogen) method developed in our previous work.^21^ In this approach, electrification is introduced by adding an excess electronic charge *q* = −*eN*_*e*_, which is counterbalanced by uniformly modifying the core charges of all *N*_H_ hydrogen atoms in the water layer by Δ*q*_H_ = *eN*_*e*_/*N*_H_. This enables systematic control of the surface excess charge density, σ = *q*/*A*, where *A* is the total surface area of the symmetric slab. Because this construction avoids explicit interfacial ions, it isolates the metal–water response from ion-specific effects while retaining electrostatic screening. At the same time, the compensating countercharge distribution converges on the same time scale as the interfacial water density distribution, that is, within AIMD-accessible simulation times of approximately 30–50 ps; see Section S1 of the Supporting Information (SI) for details. The effective electrode potential corresponding to a given surface charge density σ is obtained from the difference between the Fermi level, ϵ_F_(σ), and the electrostatic reference potential in the bulk-water region, ϕ^S^(σ), ϕ_*E*_(σ) = −⟨ϵ_F_(σ)/*e*–ϕ^S^(σ) ⟩_σ_. The resulting σ–ϕ_*E*_ relationships are shown in Figure S3 of the SI. Within this framework, the differential capacitance follows directly from the microscopic simulations as $C_{\mathrm{AIMD}}=\frac{d\sigma }{d\phi_{E}}$ .

Because the DFT-PCH method distributes the compensating countercharge throughout the water layer, the simulated capacitances are most directly comparable to measurements in highly concentrated electrolytes, that is, to the Helmholtz capacitance limit.^16,19^ For Pt(111) and Au(111), we therefore compare directly with reported high-concentration measurements, namely, the data of Pajkossy et al.^16^ for Pt(111) in 0.1 M KClO_4_ and Wang et al.^19^ for Au(111) in 2 M NaClO_4_. For Cu(111) and Ag(111), comparable high-concentration data are not available, so we use Parsons–Zobel analysis^44^ to extrapolate the Helmholtz capacitance from the available measurements; see Section S2 of the SI for details. For Cu(111), possible pseudocapacitive contributions from OH adsorption were removed in this analysis.^17,45,46^ For Ag(111), we use the measurements of Valette et al.^18^ in the PF_6_^–^ electrolyte, as no comparable ClO_4_^–^ dataset without specific adsorption is available. The possible effect of the ion identity on the Helmholtz capacitance is discussed later. Overall, the agreement between the simulation and experiment in the Helmholtz limit is very good. Having established that the simulations reproduce the experimental metal-dependent trend, we next use them to dissect its microscopic origin.

### Peak Capacitance as an Interfacial Property

To rationalize the experimental Helmholtz capacitance peak near the PZC, we focus on the peak regime, which in our simulations corresponds approximately to surface charge densities of σ ≈ −10 to 20 μC cm^–2^ for each metal; see Section S3 of the SI for details. Following our previous work,^21,22^ we decompose the overall potential drop, Δϕ (σ), across the electrified metal–water interface into an electrostatic bias contribution and a water-screening contribution

2 $$\Delta \phi \left(\sigma \right)=\Delta \phi^{\mathrm{bias}}\left(\sigma \right)+\Delta \phi^{w}\left(\sigma \right)$$

Here, the electrostatic bias potential, ϕ^bias^(σ,*z*), is obtained from the difference between the planar-averaged Hartree potentials of charged and neutral single-point calculations performed on the same AIMD snapshots, ϕ^bias^(σ, z) = ϕ_Hartree_^charged^(z) – ϕ_Hartree_^neutral^(*z*), with representative profiles shown in Figure S8­(d) of the SI.

We further partition the two contributions, Δϕ^bias^(σ) and Δϕ^w^(σ), into interfacial and bulk components. Specifically, Δϕ^bias‑interface^(σ) denotes the interfacial part of the electrostatic bias response, while Δϕ^w‑interface^(σ) denotes the contribution from interfacial water. The remaining terms are collected into a bulk contribution, Δϕ^bulk^(σ); additional details are provided in Section S3 of the SI

3 $$\Delta \phi \left(\sigma \right)=\Delta \phi^{\mathrm{bias}‐\mathrm{interface}}\left(\sigma \right)+\Delta \phi^{w‐\mathrm{interface}}\left(\sigma \right)+\Delta \phi^{\mathrm{bulk}}\left(\sigma \right)$$

This partition follows the physical logic of the Gouy–Chapman–Stern picture, in which the potential drop across an electrified interface is separated into compact-layer and diffuse-layer contributions. Our previous work showed that both the interfacial water structure and the interfacial bias-potential distribution are already converged within the simulated interfacial region.^21^ For Δϕ^w‑interface^(σ), we define the interfacial water region as water within 4.5 Å of the surface for Pt(111)^47^ and Cu(111) and within 4.8 Å for Ag(111) and Au(111). These cutoffs are determined from the corresponding bilayer water density profiles, using the distributions of the water oxygen density and orientational density along the surface normal. Δϕ ^w‑ interface^(σ) is then obtained by retaining only the metal slab and interfacial water molecules from each AIMD snapshot and removing the bulk water. As detailed in Sections S3 and S5 of the SI, the analysis and conclusions put forward below are insensitive to the specific cutoff values employed.

Because the differential capacitance satisfies *C* ^–1^ = ∂ Δϕ/∂σ, this potential drop decomposition leads directly to an additive decomposition of the inverse capacitance

4 1C=∂Δϕbias‐interface∂σ︸1/Cbias‐interface(σ)+∂Δϕw‐interface∂σ︸1/Cw‐interface(σ)+∂Δϕbulk∂σ︸1/Cbulk(σ)=∂Δϕinterface∂σ︸1/Cinterface(σ)+∂Δϕbulk∂σ︸1/Cbulk(σ)

where in the second line, both interfacial contributions (bias interface and w-interface) to the total capacitance have been combined to *C*_interface_. Figure 2(a) shows that this *C*_interface_ is strongly correlated with the DFT-AIMD peak capacitance, *C*_max_, across the metal series. This indicates that the metal-dependent Helmholtz capacitance peak is primarily governed by the interfacial region. Consistently, the corresponding inverse capacitance analysis in Figure S7 of the SI gives a small fitted intercept of approximately 0.003 (μF cm^–2^)^−1^, indicating that the bulk contribution is minor on the scale of the present data. This is expected from the high dielectric response of bulk water, which screens the residual electrostatic potential drop in the bulk region for the present cell sizes. Thus, to first order, the bulk-water contribution is negligible and approximately system-independent.

**Figure 2:**
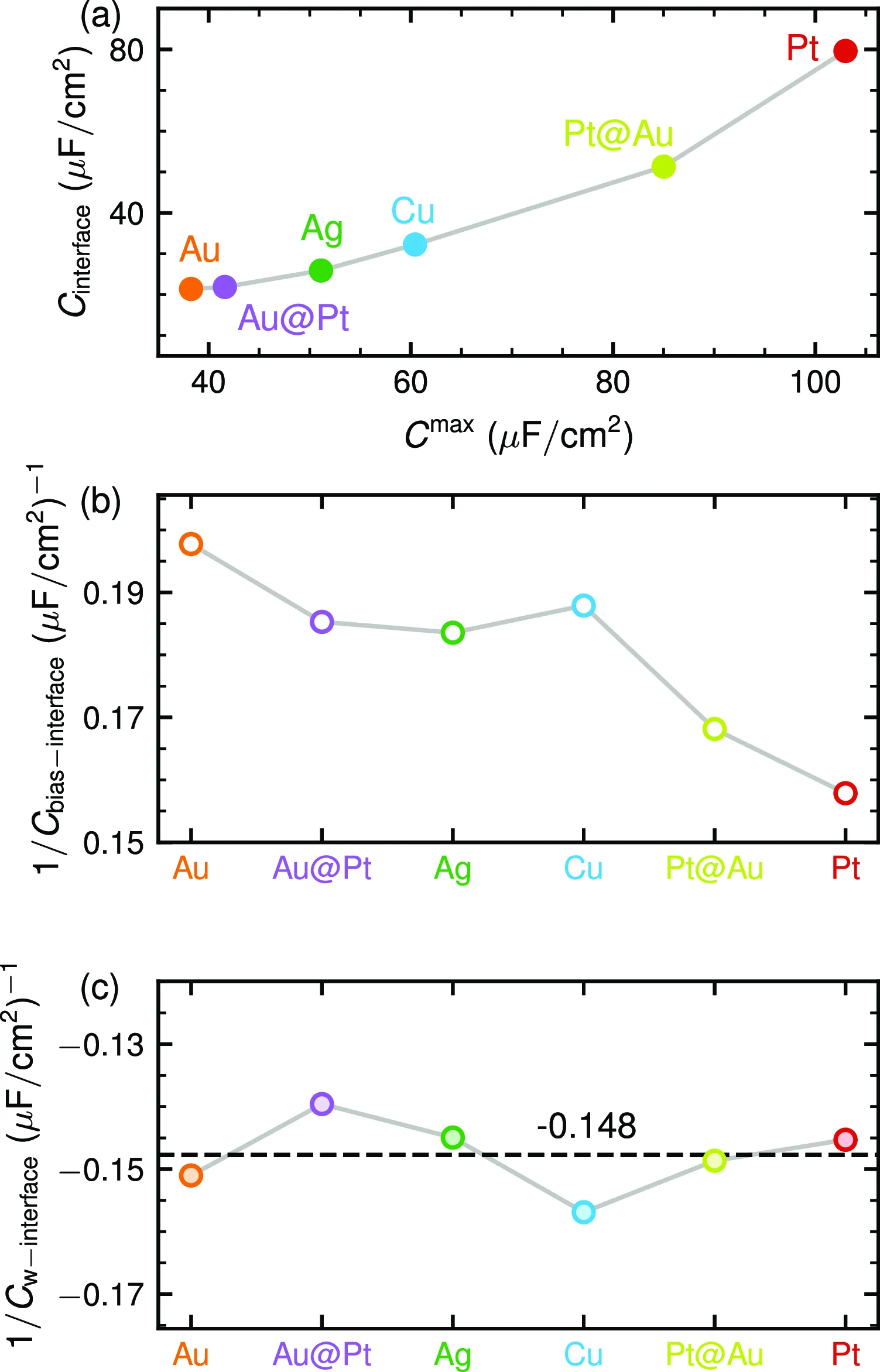
Interfacial origin of the metal-dependent Helmholtz capacitance. (a) Interfacial capacitance, *C*_interface_, correlated with the DFT-AIMD peak capacitance, *C*_max_. (b) Metal-dependent interfacial bias contribution, 1/*C*_bias‑interface_. (c) Interfacial water response contribution, 1/*C*_w‑interface_, which is weakly metal-dependent and fluctuates around an approximately constant value; the dashed line denotes its average. All quantities are evaluated in the peak regime, σ ≈ −10 to 20 μC cm^–2^, corresponding to the unshaded region in Figure 1; see Section S3 of the SI for details.

Figure 2(b,c) further resolves the interfacial inverse capacitance into interfacial bias and water-response contributions. The interfacial bias term, 1/*C*_bias‑interface_, shows a clear metal dependence, whereas the interfacial water-response term, 1/*C*_w‑interface_, varies only weakly around an approximately constant value. Thus, the metal dependence of the peak capacitance is largely encoded in the interfacial bias response. This motivates us to examine the microscopic origin of 1/*C*_bias‑interface_.

### Electrode Electron Spillover as a Driver for Metal-Dependent Helmholtz Capacitance

To identify the microscopic origin of the metal-dependent bias response, we analyze the spatially resolved bias potential, ϕ^bias^(σ,*z*), and the corresponding response charge distribution. The bias potential is governed by Poisson’s equation

5 $$\frac{\partial^{2}\phi^{\mathrm{bias}}\left(\sigma ,z\right)}{\partial z^{2}}=-\frac{\rho_{\mathrm{electrode}}\left(\sigma ,z\right)+\rho_{\mathrm{counter}}\left(\sigma ,z\right)}{\varepsilon_{0}}$$

where the total bias charge comprises the bias-induced electronic response, ρ_electrode_(σ,*z*), and the compensating countercharge, ρ_counter_(σ,*z*). The latter is the known counterbalancing charge introduced through the modified hydrogen core charges in the PCH approach.^21^ We obtain ρ_electrode_ by deriving the total bias charge from the bias-potential profile using eq 5 and subtracting the known countercharge; full details are provided in Section S3.1.1 of the SI. Here, ρ_electrode_ is used operationally to denote the bias-induced electronic response associated with electrode charging and should not be interpreted as a strict partition of electronic charge belonging exclusively to the metal.

To focus on the physically relevant interfacial component of this response, we retain ρ_electrode_ within the interfacial region and remove residual contributions from the bulk-water region beyond *z* = 5.3 Å, as detailed in the SI. In the following, ρ_electrode_(σ,*z*) denotes this interfacial electrode response. As shown exemplarily for Pt(111) in Figure 3a, it is not confined to the metal slab but extends beyond the nominal metal surface into the interfacial water region. Analogous profiles are obtained for the other metals, as shown in Figures S11 and S12 of the SI.

**Figure 3:**
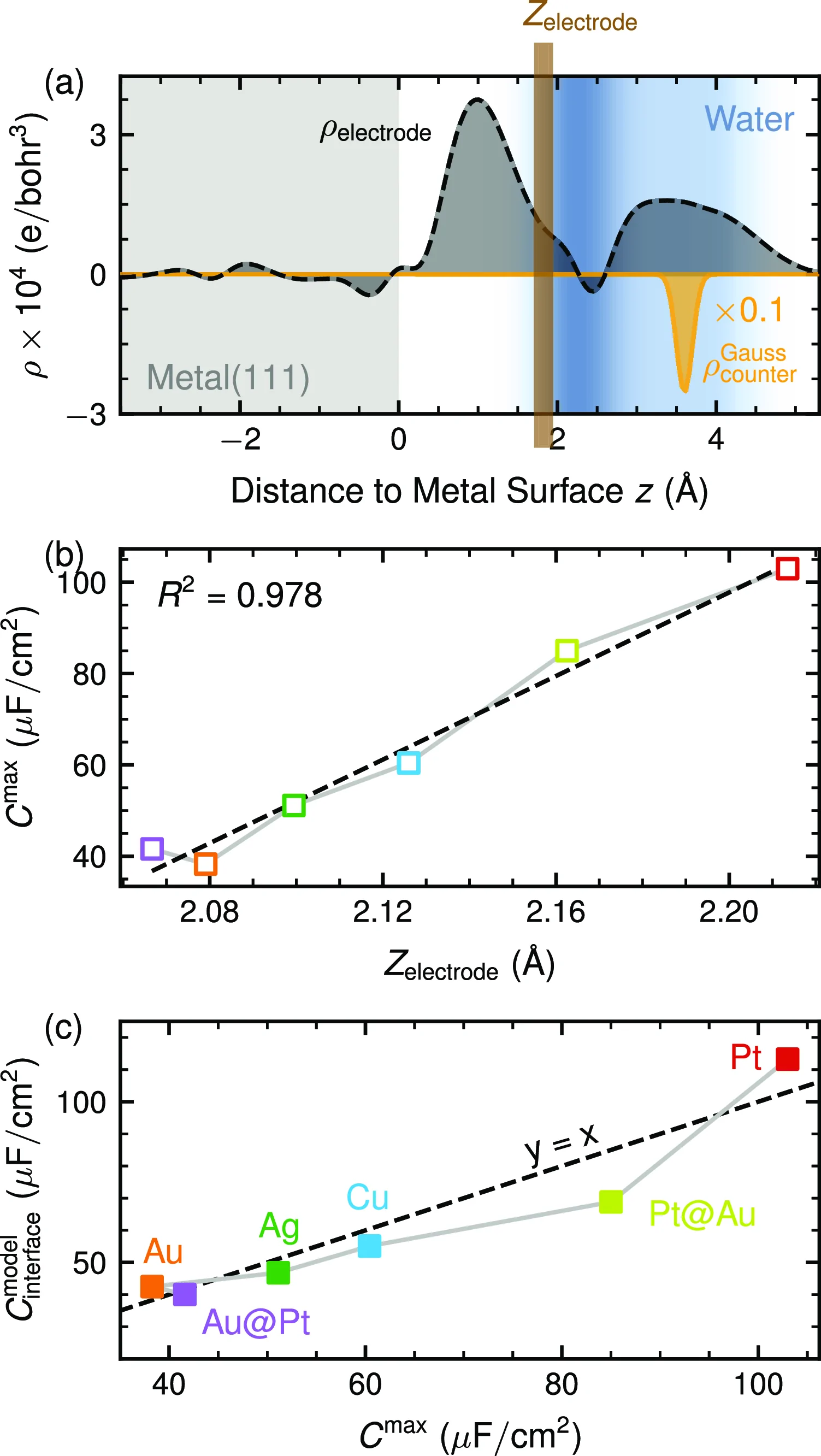
Metal-side electronic response governs the metal-dependent Helmholtz capacitance. (a) Planar-averaged interfacial electrode-response charge for Pt(111), ρ_electrode_, obtained from the bias-potential profile after subtracting the known countercharge contribution. Residual bulk-water contributions (*z* > 5.3 Å) are removed as detailed in Section S3.1.1. The Gaussian countercharge model, ρ_counter_^Gauss^, is shown in orange. Gray indicates the metal slab and blue indicates water, with darker and lighter shades corresponding to chemisorbed and nonchemisorbed water, respectively. Analogous profiles for all metals are shown in Figures S12 and S13 of the SI. (b) The extracted charge-center position, *Z*_electrode_, is strongly correlated with the DFT-AIMD peak capacitance, *C*_max_. (c) Model interfacial capacitance, *C*_interface_^model^, obtained by combining the model bias capacitance with the average interfacial water contribution from Figure 2c. The resulting *C*_interface_^model^ is compared with *C*_max_ from DFT-AIMD. The black dashed diagonal line indicates *C*_interface_^model^ = *C*_max_.

We refer to this field-induced extension of the electrode response into the water side as electron spillover. Importantly, this terminology does not imply charge transfer due to covalent metal–water bond formation but describes the redistribution of electronic density across the metal–water interface under electrification. Because this response requires an electronic structure description, it is absent in classical fixed-charge molecular dynamics. Electron spillover into interfacial water is observed across different electronic structure charging schemes^28,48–51^ and has experimental support as well.^52^ Its spatial extent, however, is strongly metal-dependent. To quantify this variation, we define the effective electrode charge center

6 $$Z_{\mathrm{electrode}}=\frac{\int z\rho_{\mathrm{electrode}}\left(z\right)dz}{\int \rho_{\mathrm{electrode}}\left(z\right)dz}$$

This quantity provides a compact electrostatic measure of how far the electrode response charge extends toward the electrolyte. As shown in Figure 3b, *Z*_electrode_ is strongly correlated with the DFT-AIMD peak capacitance, *C*_max_, across the pure-metal series. This correlation suggests the metal-dependent spillover length as the microscopic origin of the *C*_max_ trend.

To further support this interpretation, we use a simple capacitor model in which the interfacial electrode response is represented by ρ_electrode_(*z*), while the compensating charge is approximated by a Gaussian countercharge distribution, ρ_counter_^Gauss^(*z*), centered at *z* = 3.6 Å. This representative near-PZC interfacial position is consistent with explicit-ion DFT-AIMD simulations^24,27^ and experimental estimates.^53,54^ Solving eq 5 for this charge distribution yields the model bias capacitance, *C*_bias‑interface_^model^, with technical details and sensitivity tests provided in Section S3.1.4 of the SI. Since the interfacial water contribution is only weakly metal-dependent (Figure 2c), we approximate 1/*C*_w‑interface_ by its average value and combine it with the model bias term using eq 4. This yields a model interfacial capacitance, *C*_interface_^model^, which reproduces the DFT-AIMD trend in *C*_max_ (Figure 3c). This agreement supports the interpretation that the metal-dependent Helmholtz capacitance is governed primarily by electron spillover, whereas the interfacial water response acts mainly as an approximately constant background.

The descriptor *Z*_electrode_ provides an averaged electrostatic measure of this response and is linked to the local interfacial electronic polarizability that controls interfacial screening.^48^ A larger separation *d* between the effective electrode-response charge center and the countercharge produces a larger potential drop and hence a smaller capacitance, analogous to a parallel-plate capacitor, with *C* ∼ *d* ^–1^. Conversely, stronger spillover toward the interfacial water enhances screening, reduces the potential drop, and increases the capacitance.

Because *Z*_electrode_ reflects an interfacial screening response, it is not an electrode property alone. In real electrochemical double layers, the compensating charge distribution depends on the electrolyte identity and ion positions, effects that are not explicitly captured by the PCH approach.^55,56^ Such ion-specific contributions may account for residual deviations between the simulation and experiment, particularly for Ag(111) for which the experimental reference involves a different anion.

### Transferability of the Interfacial Spillover Descriptor

The analysis above establishes a microscopic model for the Helmholtz capacitance trend at metal(111)/water interfaces. Across the pure-metal series, Pt > Cu ∼ Ag > Au, the peak capacitance trend is already captured by the interfacial region response (Figures 1 and 2). Decomposing this response into electrostatic bias and water contributions shows that the trend is governed primarily by metal-side electron spillover, whereas the water screening response is only weakly metal-dependent.

This interfacial origin explains why clean-surface quantities do not recover the capacitance trend. The density of states (DOS) at the Fermi level is commonly used as a simple proxy for electronic polarizability, while the work function and occupied *d*-band center encode other aspects of the intrinsic surface electronic structure. However, when evaluated for the corresponding bare-metal surfaces, none of these quantities follows the *Z*_electrode_ trend (Section S6 of the SI). They capture the intrinsic electronic response of the isolated metal but not the interfacial charge redistribution induced by water. The relevant descriptor is therefore not a property of the metal alone but an emergent property of the electrified metal–water interface.

We next test whether this interfacial descriptor also captures capacitance changes beyond the pure-metal series, including atomistic lattice effects. For this purpose, we consider two cross-geometry systems, Pt@ Au(111) and Au@ Pt(111), in which the lattice constants of Pt and Au are interchanged. The corresponding DFT-AIMD capacitance curves are shown in Figure S4 of the SI. Pt@ Au(111) reaches a peak capacitance of approximately 80 μF cm^–2^, intermediate between those of Pt(111) and Au(111) but closer to that of Pt(111), whereas Au@ Pt(111) yields a peak capacitance of approximately 42 μF cm^–2^, close to that of Au(111).

Importantly, both cross-geometry systems follow the same *Z*_electrode_–*C*_max_ relation as the pure-metal series (Figure 3b). The model interfacial capacitance obtained from the spillover-based bias term and the average water contribution likewise remains consistent with the DFT-AIMD peak capacitances (Figure 3c). Thus, *Z*_electrode_ is not merely interpolating the elemental metal sequence but captures lattice-dependent changes in the interfacial screening response.

This transferability is important because the cross-geometry systems decouple the metal identity from lattice geometry, thereby testing a contribution that is absent from earlier work function-based correlations. Such correlations, including the Trasatti picture for simple *sp* metals,^38^ relate capacitance trends to intrinsic properties of the bare-metal surface but do not explicitly resolve atomistic lattice effects or the modification of the metal response by interfacial water. Together with the failure of clean-surface descriptors (Section S6), this confirms that the relevant physics cannot be described by properties of the metal alone; it requires an account of the collective electronic response of the explicit electrified metal–water interface.

## Conclusions

We have developed a unified microscopic framework for understanding the Helmholtz capacitance at metal(111)/water interfaces by combining extensive DFT-AIMD simulations with a physically transparent potential drop decomposition. The simulations reproduce the long-established experimental peak capacitance trend, Pt > Cu ∼ Ag > Au,^10,13,14,32^ and identify its microscopic origin as interfacial electron spillover. This metal-side response, generated at the explicit metal–water interface, shifts the effective charge-center position of the electrode response and thereby controls the bias contribution to the interfacial capacitance, while the water screening response remains only weakly metal-dependent. We expect this understanding of the relative trend to be robust to the approximate treatment of electronic exchange and correlation at the present PBE-D3 level. However, the absolute magnitude of spillover and the concomitant capacitance are likely compromised by deficiencies in the simultaneous description of a metallic electrode in contact with the semiconducting electrolyte.

The picture developed here rationalizes why clean-surface descriptors, including the work function, occupied *d*-band center, and DOS at the Fermi level, do not recover the capacitance trend: they capture only intrinsic properties of the isolated metal surface but not the interfacial charge redistribution induced by water. Our *Z*_electrode_-based description further captures the capacitance variations of the cross-geometry Pt/Au systems, demonstrating that the mechanism extends beyond the pure-metal series and includes lattice-dependent changes in the interfacial screening response. Together, these findings identify electron spillover at the explicit metal–water interface as the microscopic origin of the metal dependence of the Helmholtz capacitance peak.

Our results may connect directly to the recent interpretations of anomalous Parsons–Zobel slopes in terms of interfacial correlation effects and effective image-plane physics at metal–electrolyte interfaces.^57–60^ The present ion-free DFT-AIMD results suggest that part of the effective separation between ionic charge and the metal image-charge response is likely influenced by the explicit metal–water interface properties, in particular, the metal-dependent electron spillover shifts. This logic is supported by our observed trends across the electrode metal and experimentally reported trends in anomalous Parsons–Zobel slopes.

The present results suggest several directions for future work. One is to test whether the electrode-side descriptor identified here can also rationalize capacitance trends across different surface orientations, including low-index and stepped interfaces. Such systems are attracting increasing attention, e.g., as models for a realistic electrocatalyst under working conditions, yet their interfacial capacitance remains difficult to interpret microscopically. In this context, the present metal response-based picture may offer a complementary perspective to interpretations based primarily on the interfacial water orientation or specific adsorption, including OH-related effects.^61,62^ A second question concerns the role of electrolyte identity. Comparisons between PF_6_^–^- and ClO_4_^–^-based systems indicate that the ion identity can influence the measured capacitance.^16–19^ It may contribute to the small Ag–Cu discrepancy between the experiment and simulation in Figure 1. It will therefore be important to clarify how ion-specific effects modify the interfacial charge distribution, even under nominally identical electrolyte concentrations.^13,14,27^ A third open question is how the microscopic structure and dynamics of interfacial water generate the water-side contributions identified here, a topic also being actively probed by interfacial spectroscopy.^63,64^ Addressing these issues should help complete the physical picture of electrified metal–water interfaces.

## Supplementary Material



## Notes

Detailed descriptions of the computational setup and electrified metal(111)/water interface models, determination of the electrode potential, charge distribution analysis and decomposition of the potential drop and capacitance, experimental capacitance analysis, characterization and modeling of the interfacial electrode response and electron spillover, electrostatic significance and robustness assessment of *Z*_electrode_, structural characterization of interfacial water, and assessment of bare-metal electronic descriptors (PDF)
