# Supplementary material for: Noncovalent Metal–Water Polarization Governs Helmholtz Capacitance
Source: J Am Chem Soc. 2026 Oct 24;148(43):46699–706. doi: 10.1021/jacs.6c11740 (PMC13741841; doi:10.1021/jacs.6c11740)
Supplement: Supplementary file 1 [file ja6c11740_si_001.pdf]

Supporting Information - Non-Covalent Metal–Water Polarization Governs Helmholtz Capacitance

Lang Li,* Elias Diesen, Nicolas G. Hörmann, and Karsten Reuter*

Theory Department, Fritz-Haber-Institut der Max-Planck-Gesellschaft, Faradayweg 4-6, 14195 Berlin, Germany

E-mail: lli@fhi.mpg.de; reuter@fhi.mpg.de

Table of contents

S1 Computational Methods and Details **S3**

S1.1 Electrified Metal(111)/Water Interface Models S3

S1.2 Computational Setup S4

S1.3 Determination of the Electrode Potential S7

S2 Experimental Capacitance **S9**

S3 Decomposition of the Potential Drop and Total Capacitance **S12**

S3.1 Electrostatic Bias Response S14

S3.1.1 Charge Distribution Analysis and Bias Potential S14

S3.1.2 Bias Capacitance S17

S3.1.3 Electron Spillover into Water Layers S19

S3.1.4 Model Interfacial Bias Capacitance S21

S3.1.5 Origin of Ag–Cu Variation in the Interfacial Bias Capacitance S23

S3.2 Water-Screening Response S26

S4 Electrostatic Significance and Robustness of $Z_{\mathrm{electrode}}$ **S27**

S4.1 Electrostatic Significance S27

S4.2 Robustness with Respect to the Spatial Boundary S28

S4.3 Numerical Robustness S30

S5 Structural Properties at the Electrified Metal(111)–Water Interface **S32**

S6 Bare-Metal Descriptors Fail to Capture Interfacial Spillover **S34**

S1 Computational Methods and Details

S1.1 Electrified Metal(111)/Water Interface Models

Table S1: Summary of the computational models used for the electrified Metal(111)/water interfaces, together with the calculated work functions (Φ_M) of the corresponding clean Metal(111) surfaces. Experimental reference values[1–4] are given in parentheses.

System	Metal	Supercell	Layers	Atoms	a/Å	Φ_M/eV	H_2O	Cell dimensions/Å^3
Pt	Pt	3×4	4	48	3.98	5.5(5.9)	50	8.443×9.749×27.894
Pt@Au	Pt	4×4	6	96	4.15	5.4	69	11.738×10.165×32.980
Cu	Cu	4×4	6	96	3.60	5.3(4.9)	51	10.182×8.818×31.392
Ag	Ag	4×4	6	96	4.09	4.6(4.5)	67	11.568×10.018×32.956
Au@Pt	Au	3×4	4	48	3.98	5.4	50	8.443×9.749×27.894
Au	Au	4×4	6	96	4.15	5.2(5.3)	69	11.738×10.165×32.980

The Metal(111)/water interfaces were modeled using rectangular periodic slab supercells, as summarized in Table S1. For each metal, the slab thickness, lateral cell size, and number of water molecules were chosen to yield comparable interface models while maintaining an approximately bulk-like water density of $\sim 1\,\mathrm{g\,cm^{-3}}$. A vacuum region of 21 Å was introduced along the surface-normal direction to suppress interactions between periodic images. Differences in cell dimensions arise from the different lattice constants and corresponding supercell constructions of the metal substrates. The calculated work functions are in reasonable agreement with experimental reference values.[1–4]

Electrification was introduced by adding or removing N_e excess electrons, corresponding to a total charge $q = \pm eN_e$. Here, positive q denotes electron removal from the metal. The excess charge is predominantly localized at the metal surface, which defines the nominal surface charge density $\sigma = q/A$, where A is the total surface area of the symmetric slab. Varying N_e therefore provides systematic control over σ, while the corresponding electrode potential is obtained a posteriori from the DFT-MD simulations. The accessible surface-charge ranges are listed in Table S2. Details of the DFT-MD setup are given in Section S1.2.

Table S2: Surface charge densities (μC cm^{-2}) corresponding to different numbers of excess electrons for the electrified Metal(111)/water interface models. A denotes the total surface area of the symmetric slab (Table S1).

System	$-4e/A$	$-3e/A$	$-2e/A$	$-1e/A$	$0e/A$	$1e/A$	$2e/A$	$3e/A$	$4e/A$
Pt	−38.93	−29.20	−19.47	−9.73	0.00	9.73	19.47	29.20	38.93
Pt@Au		−20.14	−13.43	−6.71	0.00	6.71	13.43	20.14	
Cu		−26.77	−17.84	−8.92	0.00	8.92	17.84	26.77	
Ag	−27.65	−20.74	−13.82	−6.91	0.00	6.91	13.82	20.74	27.65
Au@Pt		−29.20	−19.47	−9.73	0.00	9.73	19.47	29.20	
Au	−26.85	−20.14	−13.43	−6.71	0.00	6.71	13.43	20.14	26.85

Overall charge neutrality was maintained by distributing the compensating charge over the hydrogen atoms of the water layer, $\Delta q_{\mathrm{H}} = -q/N_{\mathrm{H}}$ implemented through a core correction such that the total hydrogen core charge becomes $q_{\mathrm{H-core}} = \Delta q_{\mathrm{H}} + e$. All simulation cells therefore remain globally charge neutral. Hirshfeld population analysis in our previous work[5] showed that charge transfer between water and Pt predominantly proceeds through oxygen over the full charging range considered here, consistent with previous literature.[6–8] To avoid perturbing this intrinsic metal–water charge-transfer channel, the compensating charge was therefore assigned exclusively to the hydrogen atoms.

This construction avoids explicit counterions and isolates the effect of surface charging on interfacial water without changing the chemical composition of the interface. All models contain two symmetric interfaces, such that the net dipole cancels, as described previously.[5,9] In earlier work, we also showed that the resulting Pt(111) interfacial structure and capacitance are consistent with previous theory[10,11] and experiment.[12]

S1.2 Computational Setup

All DFT-AIMD simulations were performed with the CP2K/Quickstep package,[13] which uses a mixed Gaussian and plane-wave representation of the electronic structure. Valence electrons were treated explicitly, namely Pt 5d6s, Cu 3d4s, Ag 4d5s, Au 5d6s, O 2s2p, and H 1s, while core electrons were represented by Goedecker–Teter–Hutter pseudopotentials (O: GTH-

PBE-q6, H: GTH-PBE-q1, Pt: GTH-PBE-q10, Cu, Ag, and Au: GTH-PBE-q11).[14,15] Gaussian basis sets were of double-ζ quality with one set of polarization functions (Cu, Ag, Au, O, and H: DZVP-MOLOPT-SR-GTH; Pt: DZVP-A5-Q10-323-MOL-T1-DERIVED_SET-1),[16] and the plane-wave cutoff was set to 400 Ry. The compensating charge on the hydrogen atoms was implemented by adjusting the effective nuclear charge within the standard GTH-PBE-q1 framework. For a system with N_e excess electrons and N_{H} hydrogen atoms, the resulting core charge on each hydrogen atom becomes $1 - \frac{N_e}{N_{\mathrm{H}}}$. This construction enables different charge states to be simulated without modifying the pseudopotential files directly.

Exchange–correlation effects were described with the Perdew–Burke–Ernzerhof functional,[17] and dispersion interactions were included through the Grimme D3 correction.[18] Owing to the large simulation cells, only the Γ point was used for Brillouin-zone sampling. To verify structural convergence, we also performed DFT-AIMD simulations for Pt(111)/water in enlarged (6×6) surface cells, as commonly used in simulations of water adlayers on metal surfaces.[19] As shown in Fig. S7 of Ref. 5 and Sec. S4 of Ref. 9, the interfacial water structure is fully converged with respect to cell size. This is the key structural quantity controlling the capacitance contribution discussed in the main text.

Interface structures were sampled using second-generation Car–Parrinello molecular dynamics (SGCP-MD)[20,21] at a target temperature of 330 K. The correction step employed five iterations of orbital-transformation optimization,[22] and the integration time step was 0.5 fs. The Langevin friction coefficient γ_{L} was set to $0.001\,\mathrm{fs}^{-1}$, while the intrinsic friction coefficient γ_{D} was set to $5 \times 10^{-5}\,\mathrm{fs}^{-1}$ for Pt, Cu, Ag, and Au, and to $2.2 \times 10^{-4}\,\mathrm{fs}^{-1}$ for water; further details are given in Ref. 20. For Ag and Au, we also tested $\gamma_{\mathrm{D}} = 9 \times 10^{-5}\,\mathrm{fs}^{-1}$ under otherwise identical conditions and found no significant changes in either the interfacial water structure or the capacitance.

The water region at the PZC was first pre-equilibrated for approximately 1 ns by classical MD using the SPC/E water model,[23] yielding a water density of approximately $1\,\mathrm{g\,cm}^{-3}$. Each subsequent DFT-AIMD simulation then comprised an additional equilibration period

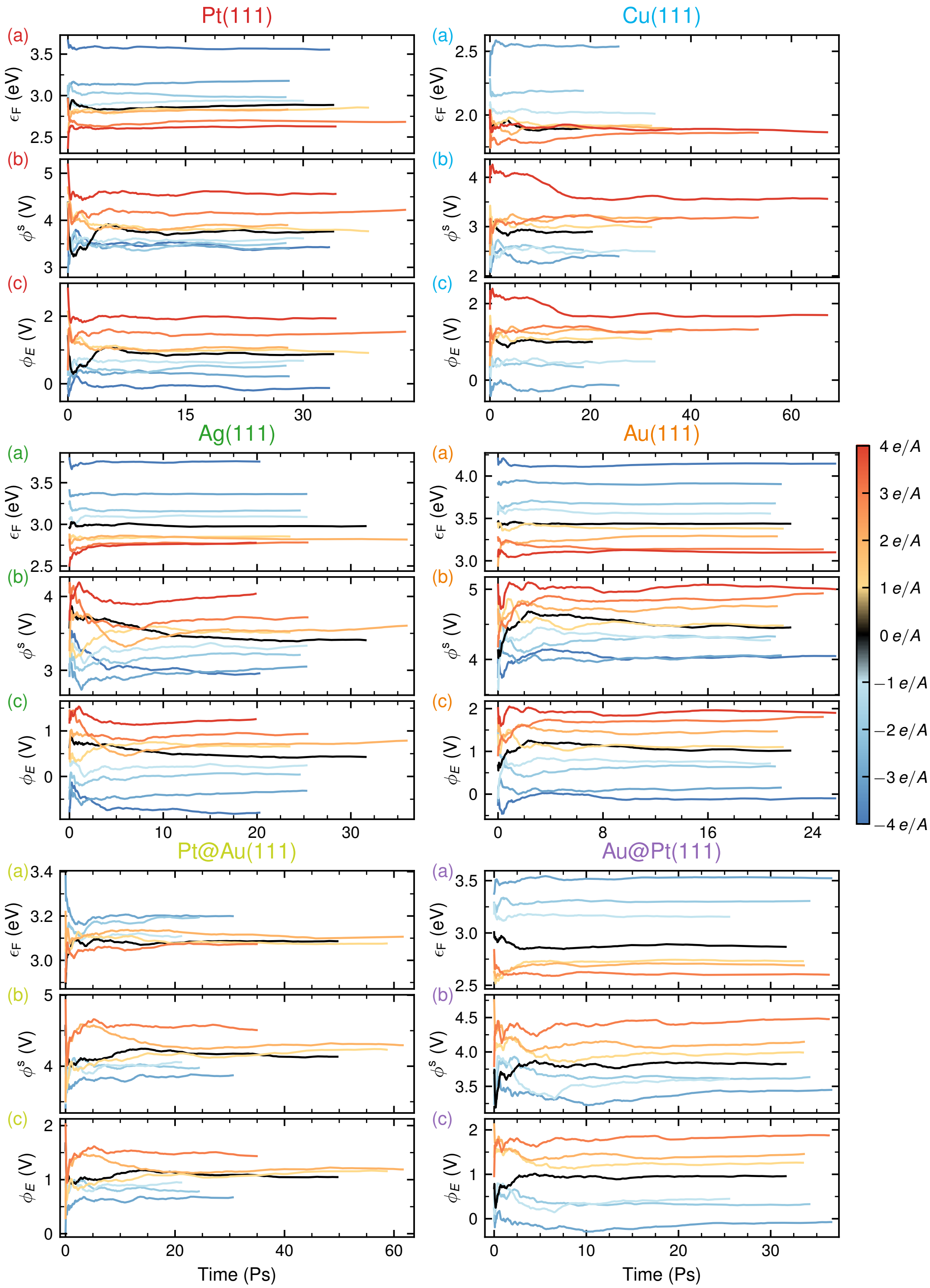

Fig. S1: Time-cumulative averages of the Fermi energy $\epsilon_{\rm F}(\sigma)$ (a), the Hartree potential in bulk water $\phi^{\rm S}(\sigma)$ (b), and the absolute electrode potential $\phi_E(\sigma)$ (c) from DFT-AIMD simulations of electrified metal/water interface models at different surface charge densities. The surface charge density is reported as $\pm eN_e/A$; see Table S2 for details.

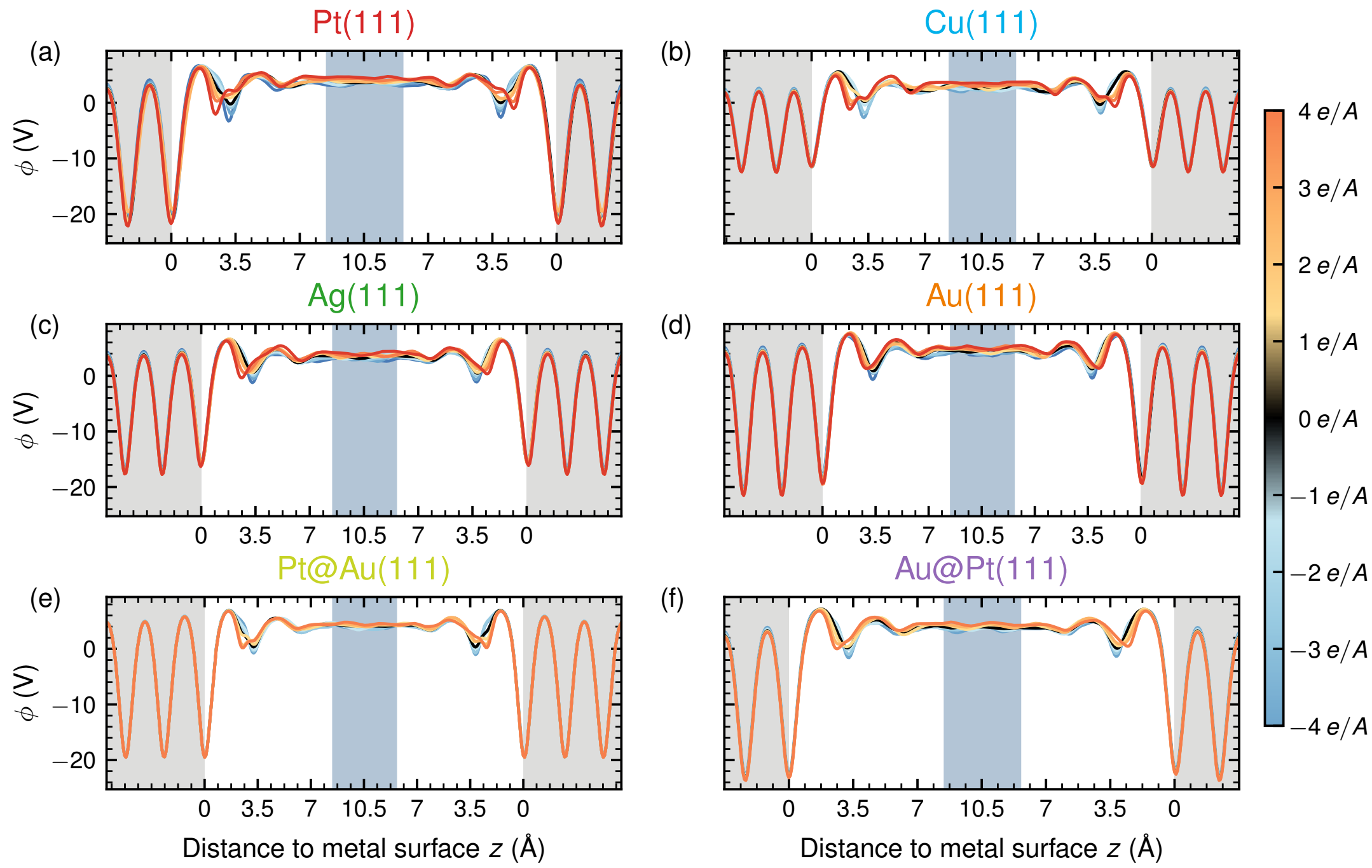

Fig. S2: Planar-averaged electrostatic potential profiles, $\phi(\sigma, z)$, along the surface-normal direction for the charged metal/water interface models. The dark-blue shaded region denotes the solvent-reference window used to determine $\phi^S(\sigma)$. Located more than 8 Å from the metal surface, this region exhibits an approximately constant potential for the charge states used to determine the electrode potential.

of approximately 5 ps (10,000 steps), followed by a production run exceeding 20 ps. During DFT-AIMD, the inner two metal layers were fixed for Pt, Cu, Ag, and Au. For Pt@Au and Au@Pt, all slab atoms were fixed to preserve the imposed lattice constant.

S1.3 Determination of the Electrode Potential

The absolute electrode potential is defined as

$$e\phi_{\text{abs}}(\sigma) = e\phi^{\text{S}}(\sigma) - \epsilon_{\text{F}}(\sigma),$$

where $\epsilon_{\text{F}}(\sigma)$ is the Fermi energy of the interface and $e\phi^{\text{S}}(\sigma)$ is the electrostatic potential in the bulk-water region, where the net charge is effectively screened. For each DFT-AIMD snapshot, the electrostatic potential was averaged over the xy plane to obtain the planar-averaged

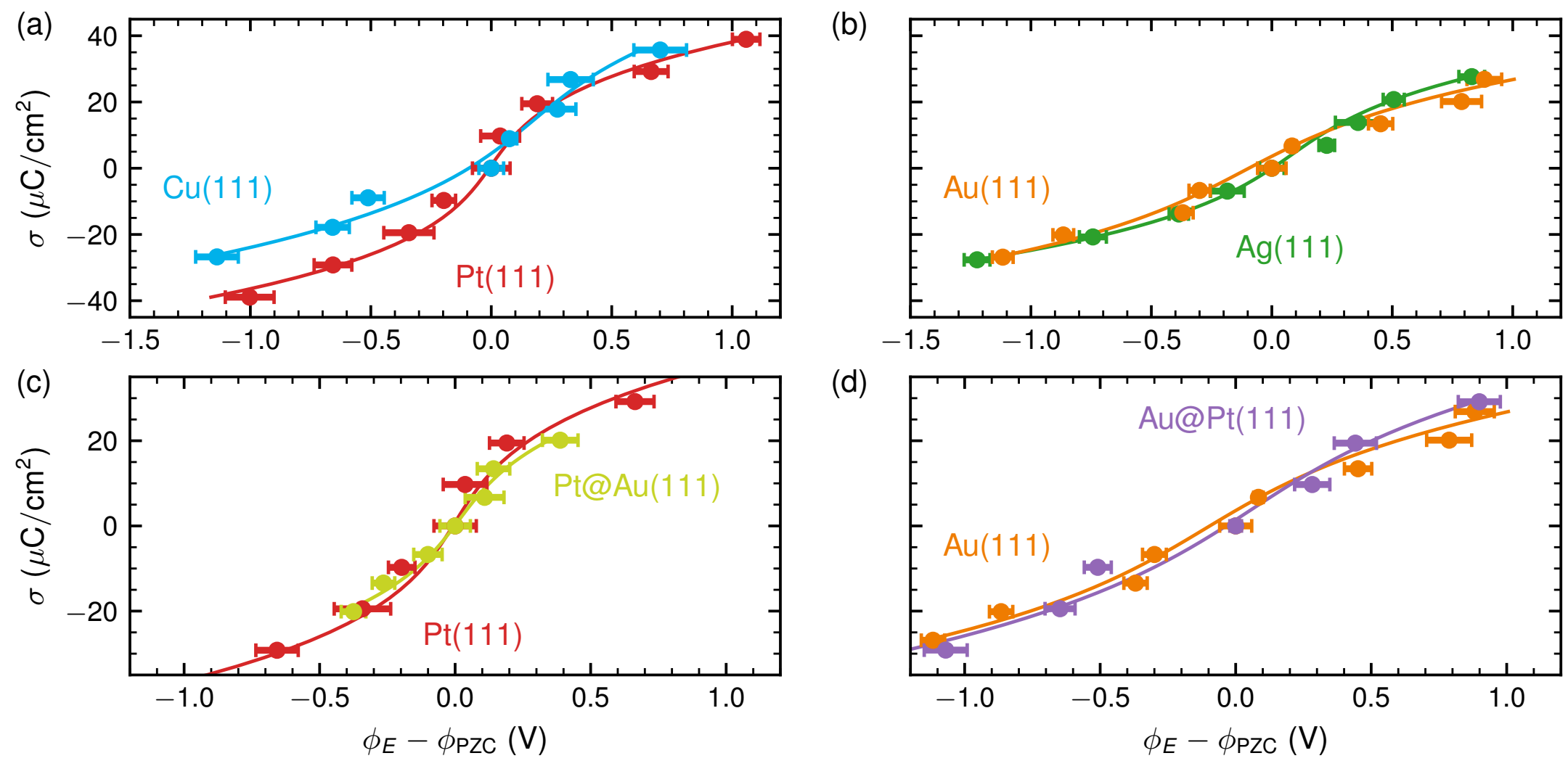

Fig. S3: Surface charge density σ as a function of electrode potential relative to the potential of zero charge, $\phi_E - \phi_{\mathrm{PZC}}$. Solid lines denote univariate spline fits used to compute the differential capacitances shown in Fig. 1 of the main text and Fig. S4.

profile $\phi(\sigma, z)$. The bulk-water reference $\phi^{\mathrm{S}}(\sigma)$ was then obtained from the approximately constant plateau region of $\phi(\sigma, z)$ in the solvent (Fig. S2). Final values of $\epsilon_{\mathrm{F}}(\sigma)$ and $\phi^{\mathrm{S}}(\sigma)$ were obtained by averaging over snapshots sampled every 50 MD steps for analysis. This sampling interval does not reduce the cost of the underlying DFT-AIMD trajectories, for which electronic-structure forces are evaluated at every MD step. Time-cumulative averages of $\epsilon_{\mathrm{F}}(\sigma)$ and $e\phi^{\mathrm{S}}(\sigma)$ are provided in Fig. S1, confirming convergence within 0.1 eV for trajectories longer than 20 ps. The corresponding $\phi_{\mathrm{abs}}(\sigma)$ values were evaluated for each trajectory. Statistical errors were estimated by dividing each trajectory into five equal blocks and computing the standard error of the mean from the block-averaged electrode potentials. These errors are shown as error bars in Fig. S3. To analyze the response to electrode polarization, all potentials were referenced to the potential of zero charge (PZC),

$$\phi_E(\sigma) - \phi_{\mathrm{PZC}} = \phi_{\mathrm{abs}}(\sigma) - \phi_{\mathrm{abs}}(\sigma = 0).$$

The electrostatic properties of the EDL were then analyzed as functions of $\phi_E - \phi_{\mathrm{PZC}}$ and σ, as shown in Fig. S3. The $\sigma \sim (\phi_E - \phi_{\mathrm{PZC}})$ relations were fitted using smooth univariate

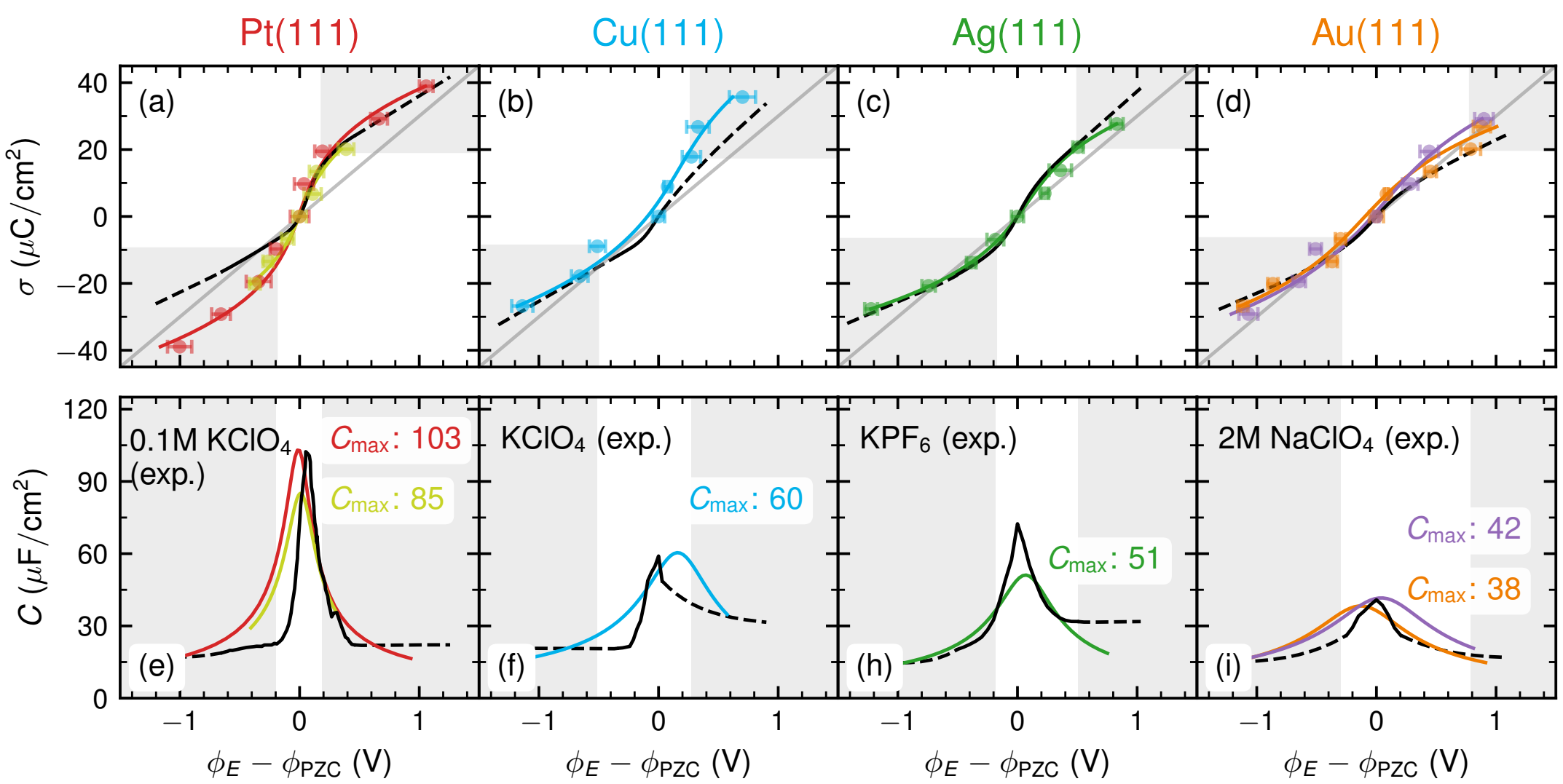

Fig. S4: Differential capacitance of electrified noble-metal/water interfaces. (a–d) Surface charge density σ as a function of electrode potential relative to the potential of zero charge, $\phi_E - \phi_{\mathrm{PZC}}$, for Pt(111), Cu(111), Ag(111), and Au(111). (e–i) Corresponding differential capacitance C, compared with experiment (black lines): Pt(111) in 0.1 M $KClO_4$,[12] Cu(111) in aqueous $KClO_4$,[24] Ag(111) in aqueous KPF_6,[25] and Au(111) in 2 M $NaClO_4$.[26] Copyright 2001, 1985, and 1989 Elsevier for Refs. 12, 24, and 25, respectively; Copyright 2025 by the American Physical Society for Ref. 26. For Cu(111) and Ag(111), experimental curves are shown after Parsons–Zobel analysis to estimate the Helmholtz capacitance (Section S2). DFT-AIMD results are shown as colored curves: Pt, red; Cu, blue; Ag, green; Au, orange; Pt@Au(111), light green; and Au@Pt(111), purple. Labels indicate the peak capacitances $C_{\max}$ extracted from DFT-AIMD.

splines, from which the differential capacitance curves in Fig. 1 of the main text and Fig. S4 were obtained.

S2 Experimental Capacitance

The experimentally measured double-layer capacitance, C_{dl}, is commonly interpreted within the Gouy–Chapman–Stern (GCS) framework, in which the total capacitance is written as a series combination of the compact-layer (Helmholtz) capacitance, C_{H}, and the diffuse-layer capacitance, C_{GC}:[27]

$$\frac{1}{C_{\mathrm{dl}}} = \frac{1}{C_{\mathrm{H}}} + \frac{1}{C_{\mathrm{GC}}}.$$

In concentrated electrolytes, C_{GC} is typically large, so that C_{dl} approaches the Helmholtz-dominated limit. Within this picture, C_{H} is often treated as only weakly dependent on electrolyte concentration, although the extent to which it is modified by ion identity remains under debate.[6,28]

For a symmetric $z\!:\!z$ electrolyte, the Gouy–Chapman differential capacitance is

$$C_{\mathrm{GC}} = \epsilon\epsilon_0\kappa \cosh\left(\frac{ze\phi}{2k_{\mathrm{B}}T}\right), \tag{S1}$$

with

$$\kappa = \sqrt{\frac{2z^2e^2c}{\epsilon\epsilon_0 k_{\mathrm{B}}T}},$$

where ϵ is the dielectric constant, ϵ_0 the vacuum permittivity, κ^{-1} the Debye length, ϕ the electrode potential relative to the potential of zero charge, c the bulk electrolyte concentration, e the elementary charge, k_{B} the Boltzmann constant, and T is the temperature. At $\phi \to 0$, Eq. S1 reduces to $C_{\mathrm{GC}}^{\mathrm{PZC}} = \epsilon\epsilon_0\kappa$.

For Pt(111)[12] and Au(111),[26] literature data are available under sufficiently concentrated conditions that the measured capacitance is already close to the Helmholtz-dominated peak response and can therefore be compared directly with our simulations (Fig. 1 of the main text). For Cu(111)[24,29,30] and Ag(111),[25] by contrast, the available literature more often reports broader hump-shaped capacitance curves, for which the compact-layer contribution must be estimated separately. For Ag(111), the nearly symmetric hump observed in KPF_6 is consistent with the absence of strong pseudocapacitive contributions over the corresponding potential window, Fig. S5b. For Cu(111), however, OH adsorption and related surface processes can contribute substantial pseudocapacitance, particularly in low-frequency measurements.[29,30] We therefore selected Cu(111) data measured at relatively low pH and in a weakly specifically adsorbing electrolyte, namely $NaClO_4$,[24] as shown in Fig. S5a

When specific adsorption and pseudocapacitive contributions are small, the GCS series relation can be analyzed using the Parsons–Zobel representation,[31] in which $1/C_{\mathrm{dl}}$ is plotted

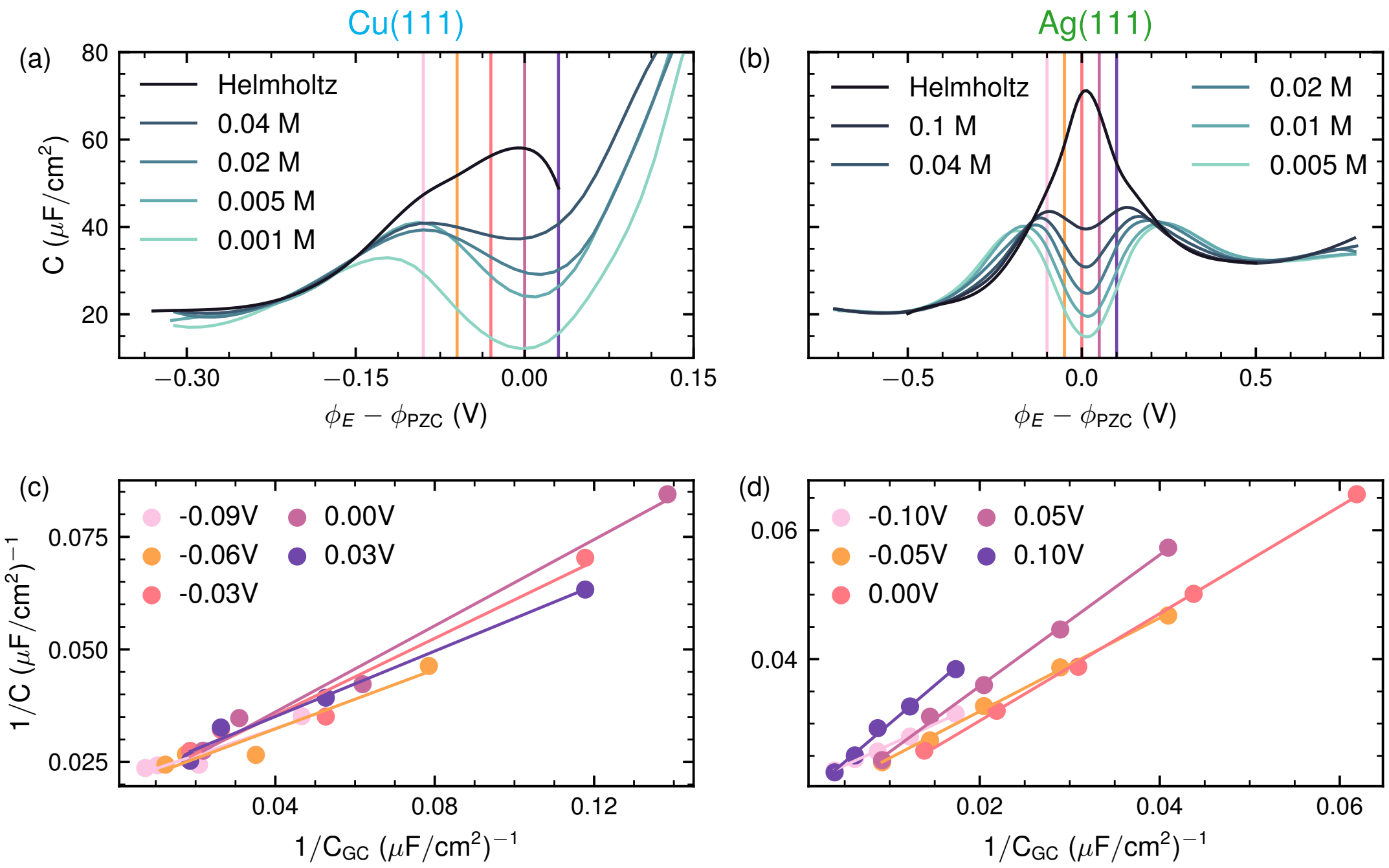

Fig. S5: Experimental capacitance analysis for Cu(111)[24] and Ag(111)[25] using Parsons–Zobel extrapolation. Experimental data were adapted with permission from Refs. 24 and 25. Copyright 1985 and 1989 Elsevier, respectively. (a,b) Measured differential-capacitance curves at different electrolyte concentrations, together with the extrapolated Helmholtz capacitance. (c,d) Corresponding Parsons–Zobel plots of $1/C_{\mathrm{dl}}$ versus $1/C_{\mathrm{GC}}$ at selected electrode potentials; the compact-layer capacitance is obtained from the intercept.

against $1/C_{\mathrm{GC}}$. If the data are approximately linear, the intercept at $(1/C_{\mathrm{GC}}) \to 0$ gives $1/C_{\mathrm{H}}$, such that

$$C_{\mathrm{H}} = \left[\lim_{1/C_{\mathrm{GC}} \to 0} \left(\frac{1}{C_{\mathrm{dl}}} \right) \right]^{-1}.$$

This provides an empirical estimate of the compact-layer capacitance from measurements performed at different electrolyte concentrations. Deviations from linearity indicate breakdown of the simple GCS description, for example due to specific adsorption, dielectric saturation, or ion-correlation effects.[32] In the present work, Parsons–Zobel analysis is used to estimate the Helmholtz capacitance of Cu(111) and Ag(111), providing the experimental reference used in Fig. 1 of the main text and Fig. S4.

S3 Decomposition of the Potential Drop and Total Capacitance

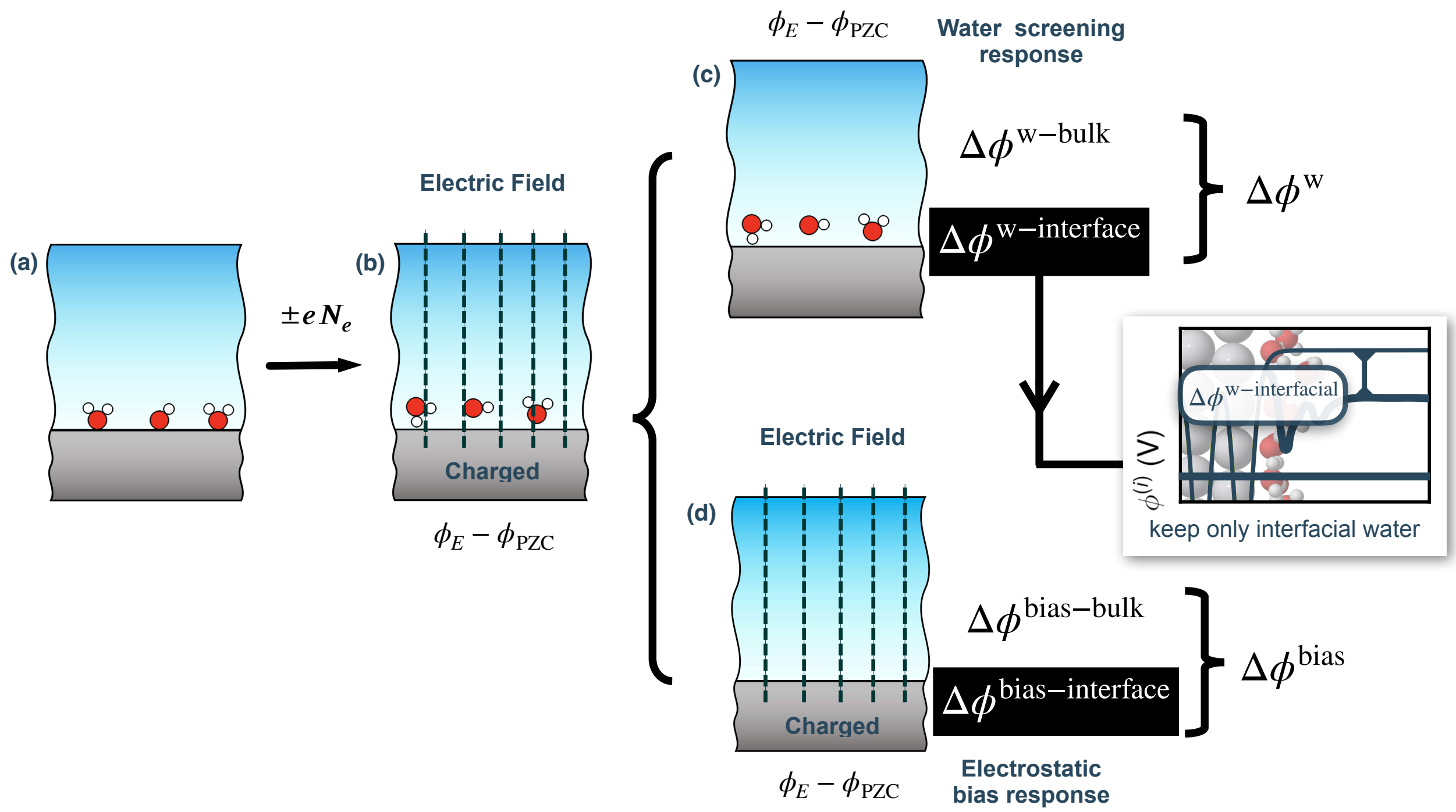

Fig. S6: Schematic decomposition of the total potential drop and the corresponding sub-capacitance contributions.

To elucidate the microscopic origin of the metal-dependent capacitance, we decompose the total potential drop, $\Delta\phi(\sigma)$, into physically distinct contributions (Fig. S6). Following the framework introduced in the main text,

$$\Delta\phi(\sigma) = \Delta\phi^{\text{bias}}(\sigma) + \Delta\phi^{\text{w}}(\sigma),$$

where $\Delta\phi^{\text{bias}}(\sigma)$ denotes the electrostatic bias response and $\Delta\phi^{\text{w}}(\sigma)$ the water-screening contribution. The bias term is further separated into interfacial and bulk components,

$$\Delta\phi^{\text{bias}}(\sigma) = \Delta\phi^{\text{bias-interface}}(\sigma) + \Delta\phi^{\text{bias-bulk}}(\sigma),$$

and the water contribution into bulk and interfacial parts,

$$\Delta\phi^{\text{w}}(\sigma) = \Delta\phi^{\text{w-interface}}(\sigma) + \Delta\phi^{\text{w-bulk}}(\sigma).$$

Combining these terms yields

$$\Delta\phi(\sigma) = \Delta\phi^{\text{bias-interface}}(\sigma) + \Delta\phi^{\text{w-interface}}(\sigma) + \Delta\phi^{\text{bulk}}(\sigma),$$

where $\Delta\phi^{\text{bulk}}(\sigma) = \Delta\phi^{\text{bias-bulk}}(\sigma) + \Delta\phi^{\text{w-bulk}}(\sigma)$. Previous work has shown that both the interfacial water structure and the bias-potential distribution are converged within the simulated interfacial region.[5] The total potential drop can therefore be partitioned into interfacial and bulk contributions, consistent with the compact-layer and diffuse-layer separation in the Gouy–Chapman–Stern picture.

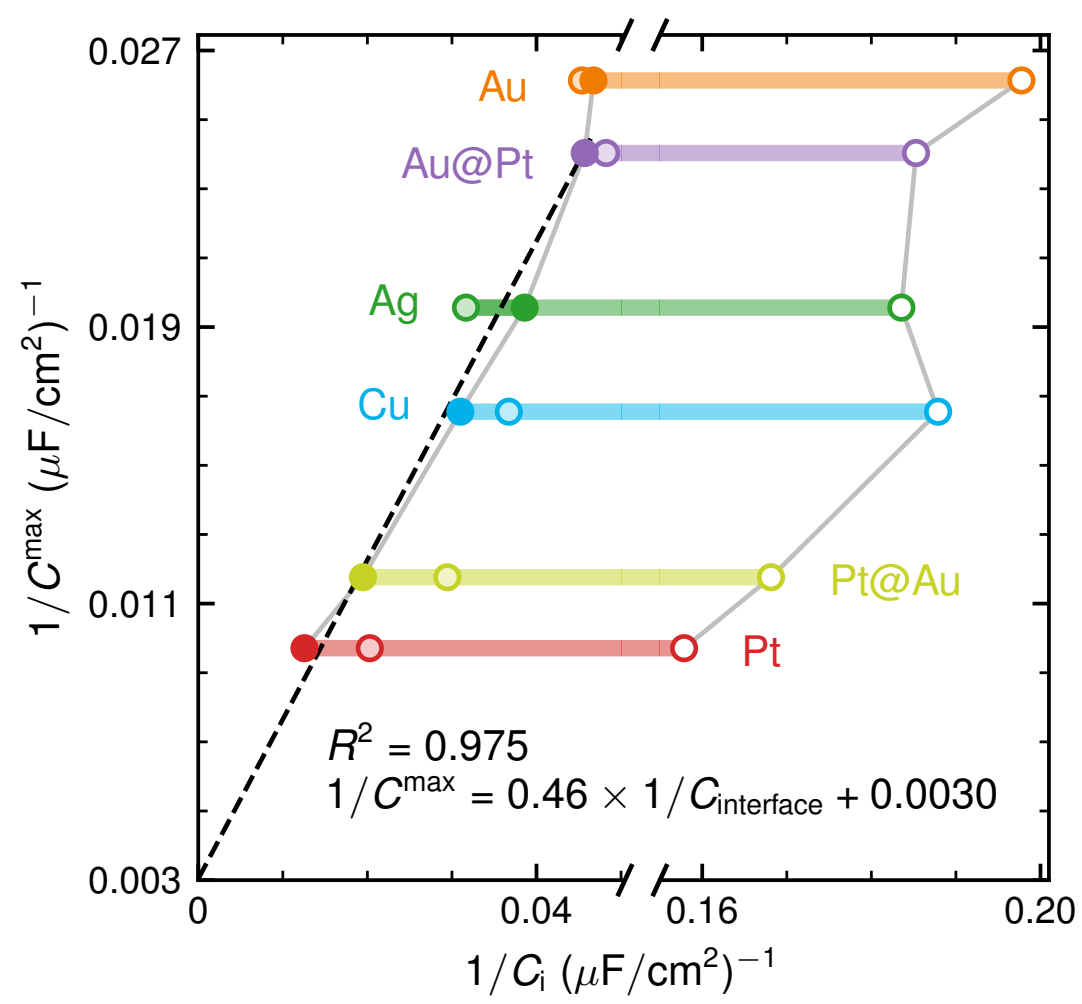

Fig. S7: Scaling between inverse peak capacitance and inverse interfacial capacitance. Inverse sub-capacitances, $1/C_i$, obtained from the potential-drop decomposition (From Figs. S9 and S16), are plotted against the inverse peak capacitance, $1/C_{\text{max}}$, extracted from DFT-AIMD in the peak regime ($\sigma \approx -10$ to 20 μC cm^{-2}). This range corresponds to the unshaded region in Fig. 1 of the main text. Open symbols denote $1/C_{\text{bias-interface}}$, shaded circles denote $1/C_{\text{w-interface}}$, and filled symbols denote $1/C_{\text{bias-interface}} + 1/C_{\text{w-interface}} = 1/C_{\text{interface}}$.

The numerical values of these decomposed potential-drop terms and the detailed extraction procedure are described below (Secs. S3.1 and S3.2). Here, we first outline the physical

picture of the decomposition. Since the differential capacitance satisfies

$$C^{-1} = \frac{\partial \Delta\phi}{\partial \sigma}, \tag{S2}$$

the inverse capacitance can be decomposed additively as

$$\frac{1}{C_{\text{tot}}} = \frac{1}{C_{\text{bias-interface}}} + \frac{1}{C_{\text{w-interface}}} + \frac{1}{C_{\text{bulk}}}. \tag{S3}$$

This decomposition separates the total interfacial response into electrostatic response, interfacial-water, and minor bulk contributions. The resulting interfacial sub-capacitance trends are shown in Fig. 2 of the main text and in Fig. S7.

S3.1 Electrostatic Bias Response

S3.1.1 Charge Distribution Analysis and Bias Potential

The central quantity in the present work is the total bias charge distribution, $\rho^{\text{bias}}_{\text{total}}(\sigma, z)$, which is composed of two primary components: the excess electron distribution, $\rho_{\text{electrode}}(\sigma, z)$, originating from the electrode, and the counter charge distribution, $\rho_{\text{counter}}(\sigma, z)$, which balances the excess electrons and ensures charge neutrality in the supercell. The excess electron distribution, $\rho_{\text{electrode}}(\sigma, z)$, represents the charge introduced into the electrode region through the addition of excess electrons (σ). In contrast, the counter charge distribution, $\rho_{\text{counter}}(\sigma, z)$ depends on the spatial distribution of hydrogen atoms.

At given excess charge σ, we evaluate average, electronic excess charge distributions $\rho_{\text{electrode}}(\sigma, z)$ by taking the ensemble average $\langle \cdot \rangle_\sigma$ using the biased MD trajectory and evaluating

$$\rho_{\text{electrode}}(\sigma, z) = \langle \rho_e(\sigma, z) - \rho_e(0, z) \rangle_\sigma \quad .$$

$\rho_e(\sigma, z)$ is the x-y averaged, self-consistent, total electron density for the biased simulation for a range of snapshots and $\rho_e(0, z)$ is the x-y averaged total electron density as obtained for

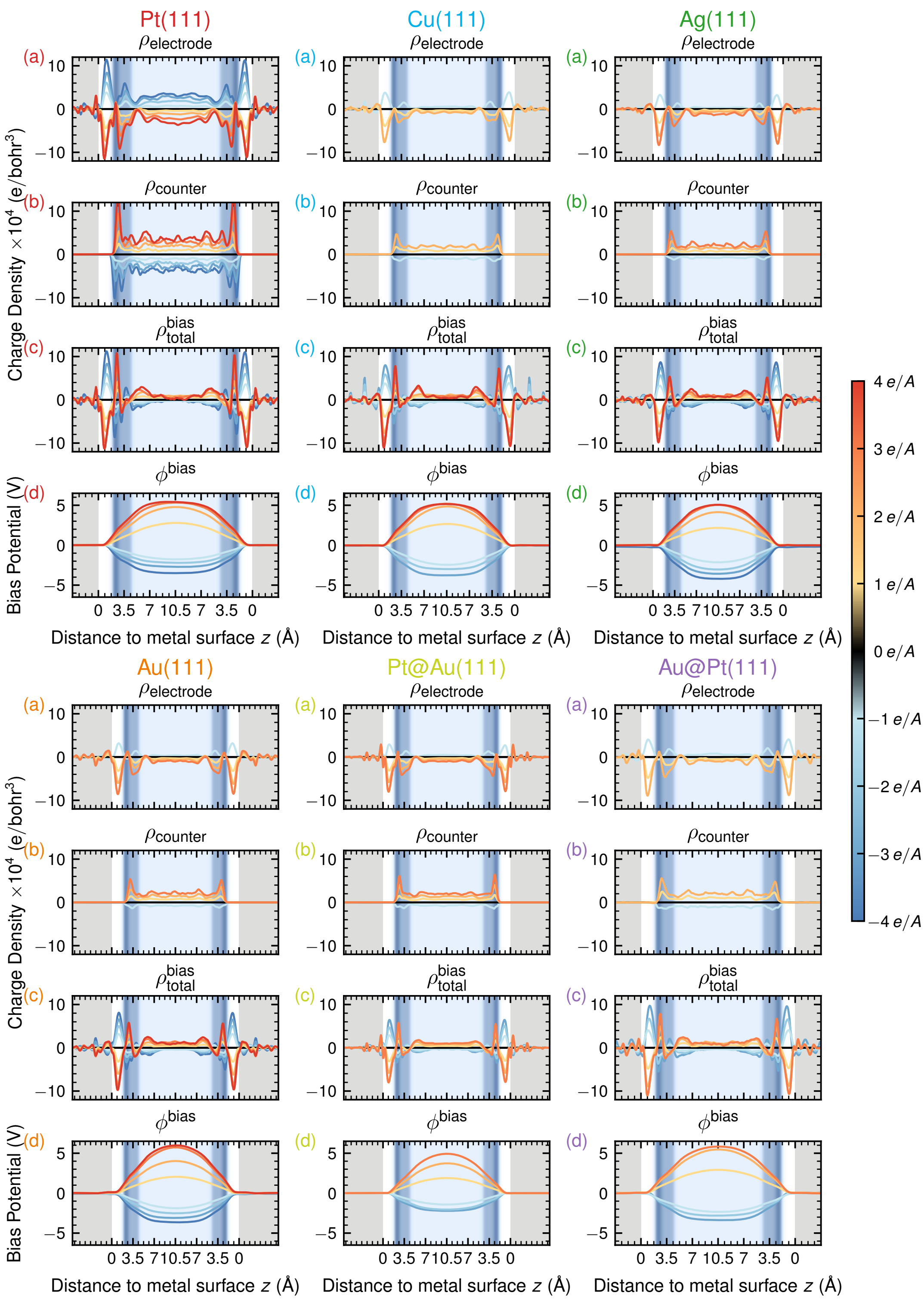

Fig. S8: Planar-averaged charge and bias-potential distributions along the surface-normal direction for the charged metal/water interfaces: (a) electrode excess charge $\rho_{\text{electrode}}(\sigma, z)$, (b) counter charge $\rho_{\text{counter}}(\sigma, z)$, (c) total bias charge $\rho^{\text{bias}}_{\text{total}}(\sigma, z)$, and (d) bias potential $\phi^{\text{bias}}(\sigma, z)$. Colors indicate different surface charge densities σ, cf. Table S2.

identical MD snapshots without present excess electrons. $\rho_e(0,z)$ is determined by reevaluation of ρ_e for each snapshot without any present bias charges. By construction, the value $\int \mathrm{d}z\,(\rho_e(\sigma,z) - \rho_e(0,z))$ thus integrates to the total number of excess charge σ present in the biased simulation, for each snapshot individually and for the average $\rho_{\mathrm{electrode}}(\sigma,z)$. $\rho_{\mathrm{electrode}}(\sigma,z)$ describes how excess electrons distribute across the electrode-electrolyte interface under varying biasing conditions and for the different biasing methods, as presented in Fig. S8a.

An analogous analysis can be performed for the countercharge density, $\rho_{\mathrm{counter}}(\sigma,z)$. As described in the main text, this contribution is directly determined by the hydrogen-density distribution in the biased systems and is reported in Fig. S8b.

As a result, one obtains the total (average) bias charge distribution

$$\rho^{\mathrm{bias}}_{\mathrm{total}}(\sigma,z) = \rho_{\mathrm{electrode}}(\sigma,z) + \rho_{\mathrm{counter}}(\sigma,z)$$

reported in Fig. S8c, which determine the system's electrostatic potential through the Poisson equation:

$$\frac{\partial^2 \phi^{\mathrm{bias}}}{\partial z^2} = -\frac{\rho^{\mathrm{bias}}_{\mathrm{total}}(z)}{\epsilon_0} \tag{S4}$$

where ϵ_0 is the permittivity of free space. The predominant localization of $\rho_{\mathrm{electrode}}(\sigma,z)$ in a confined region next to the interface, as well as suggest that the bias potential should in all cases follow an approximately parabolic behavior with

$$\phi^{\mathrm{bias}}(z) \propto \bar{\rho}^{\mathrm{bias}}_{\mathrm{total}} z^2 \tag{S5}$$

within the solution region of the simulation cells where $\bar{\rho}^{\mathrm{bias}}_{\mathrm{total}}$ is the z-averaged total charge density. In the trivial case, where no excess electrons are in water $\bar{\rho}^{\mathrm{bias}}_{\mathrm{total}}$ reduces to the (average) counter charge density $\bar{\rho}_{\mathrm{counter}}$. The expectation of an approximately parabolic biasing potential is confirmed by taking the biased MD average $\langle\cdot\rangle_\sigma$ of the system-intrinsic Hartree

potentials, determined with and without the inclusion of excess charges. This difference provides a direct measure of the parabolic biasing potential,[11] as shown in Fig. S8d. It worth noting, that (artificial) electron transfer into the bulk of the solution is observed for the DFT methods, Fig. S8a, thus reducing $\rho_{\mathrm{total}}^{\mathrm{bias}}(\sigma, z)$ in the bulk of the solution, Fig. S8c, leading to deviations from parabolic behavior as well as limiting the overall bias-induced potential drop Fig. S8d. A close inspection of Fig. S8d suggest that the DFT methods exhibit a common, intrinsic bias potential limit in the center of the water region positioned at $\sim +5$ V and ~ -2 V for positive and negative biasing directions, respectively. These limits closely coincide with the intrinsic stability window of water aka the alignment of water valence and conduction band values (see Fig. 5 in Ref. 33 for the band alignment in AIMD and Fig. 3 in Ref. 34) for the electronic stability window of static interfacial water). Hence, the observation in Fig. S8d of bias potential limits in DFT are likely not an artifact of our counter charging methods but rather a fundamental constraint due to the intrinsic electronic structure properties of water (see also a related discussion in Section S5 of the SI of our previous work Ref. 5).

S3.1.2 Bias Capacitance

From the profiles in Fig. S8d, we define the electrostatic bias-potential drop as

$$\Delta\phi^{\mathrm{bias}}(\sigma; z_{\mathrm{ref}}) = \phi^{\mathrm{bias}}(\sigma, z_{\mathrm{ref}}) - \phi^{\mathrm{bias}}(\sigma, -\mathrm{slab}),$$

where $z = -\mathrm{slab}$ denotes the electrode-side reference plane. Choosing z_{ref} in the bulk-water region yields the full-cell bias-potential drop, $\Delta\phi^{\mathrm{bias}}(\sigma)$, whereas $z_{\mathrm{ref}} = 5.3\,\text{Å}$ defines the interfacial bias-potential drop, $\Delta\phi^{\text{bias-interface}}(\sigma)$. The reference position, $z_{\mathrm{ref}} = 5.3$ Å, is chosen consistently with the electrode-response analysis shown in Fig. S11 and Fig. S9 of Ref. 9.

The resulting $\Delta\phi^{\mathrm{bias}}(\sigma)$ and $\Delta\phi^{\text{bias-interface}}(\sigma)$ curves are shown in Fig. S9. In both cases, the relation between bias-potential drop and surface charge density is not described by a

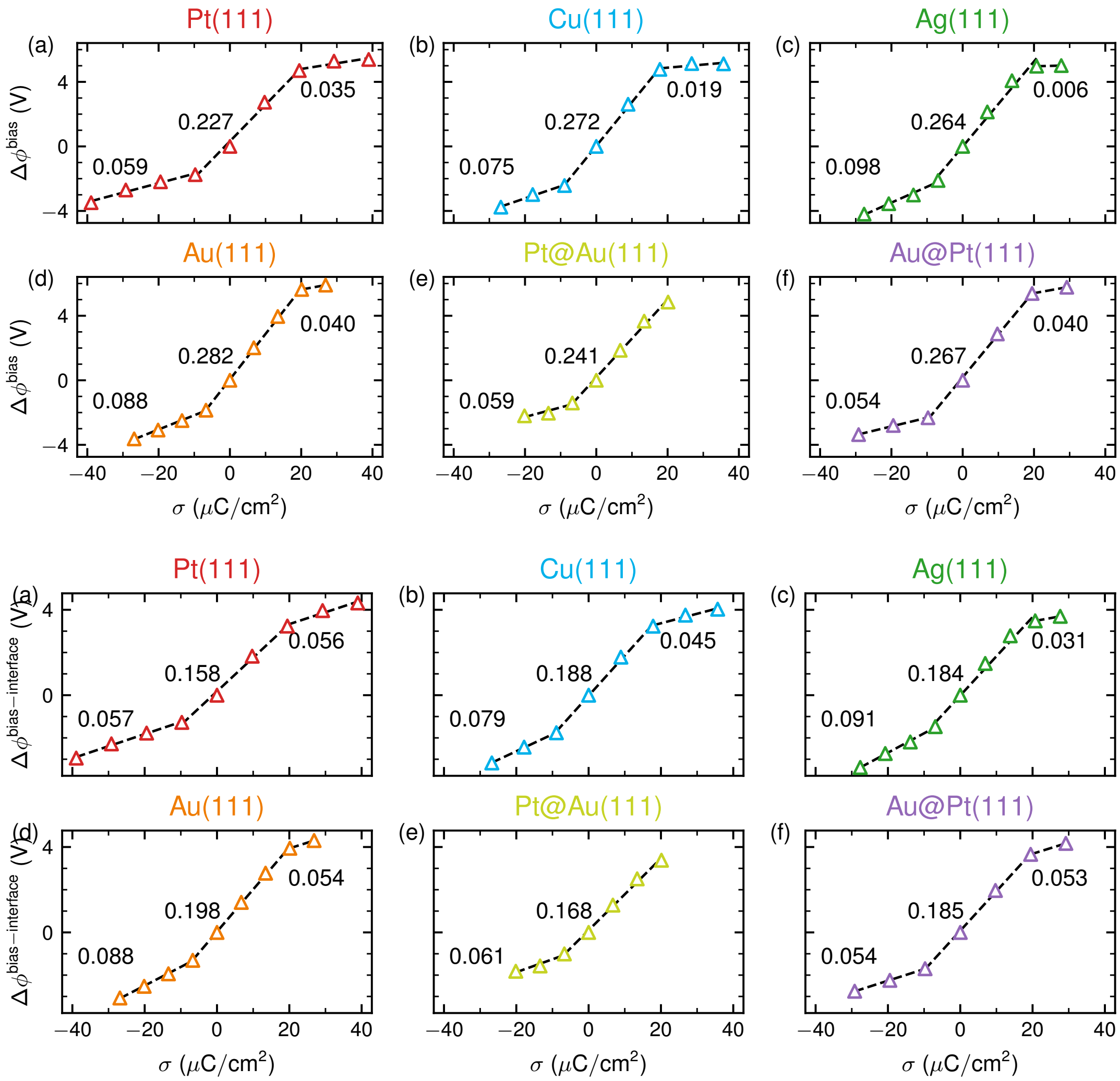

Fig. S9: Electrostatic bias-potential drops $\Delta\phi^{\text{bias}}(\sigma)$ (top) and $\Delta\phi^{\text{bias-interface}}(\sigma)$ (bottom) as functions of surface charge density σ for different metals. $\Delta\phi^{\text{bias}}(\sigma)$ is defined using the bulk-water region as the reference, whereas $\Delta\phi^{\text{bias-interface}}(\sigma)$ uses the interfacial reference position at $z = 5.3\,\text{Å}$. Dashed lines are guides to the eye. Numbers in each panel indicate the inverse capacitance obtained from the slope in the different charging regimes.

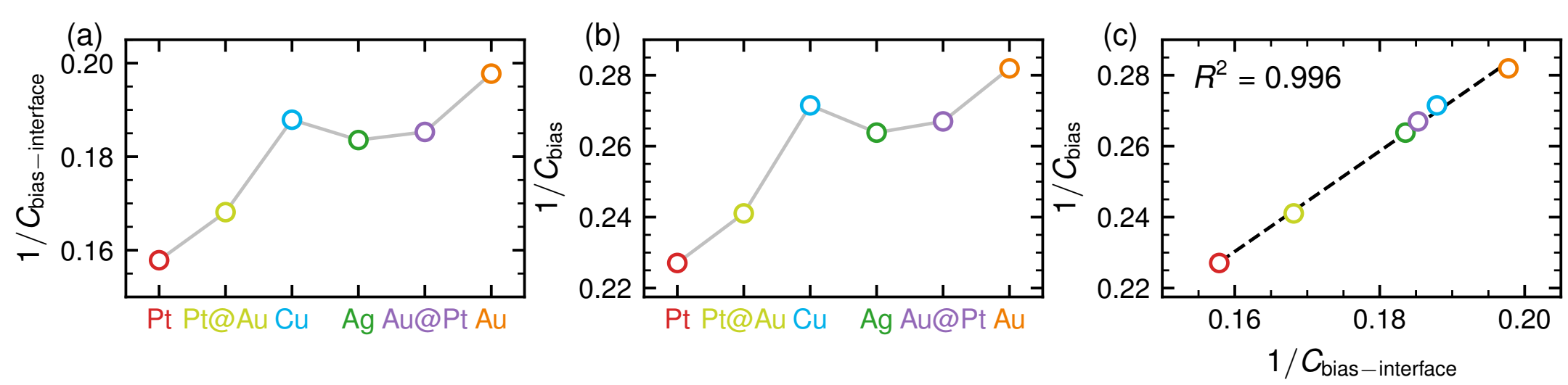

Fig. S10: Comparison of inverse bias capacitances extracted from the central charging regime: (a) $1/C_{\text{bias}}$, (b) $1/C_{\text{bias-interface}}$, and (c) correlation between the two quantities. Both definitions yield the same metal-dependent trend.

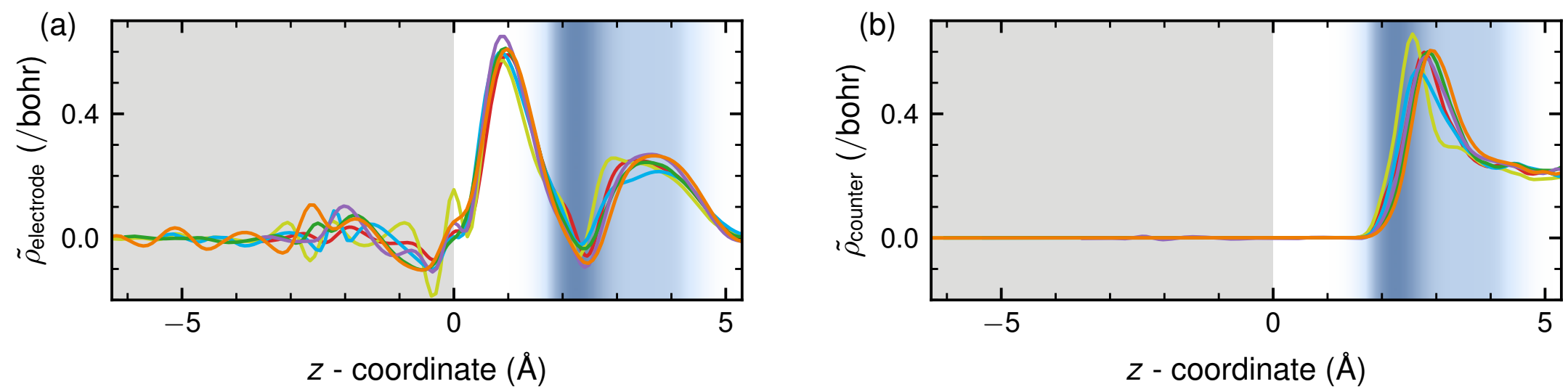

Fig. S11: Normalized electrode and countercharge response profiles in the interfacial region for the different metals, derived from Fig. S8. The profiles are normalized by their integrated values up to $z = 5.3$ Å.

single linear slope over the full charging range. Instead, three charging regimes can be identified, each characterized by a distinct inverse bias capacitance according to Eq. S2. This nonlinear behavior is consistent with the peak-shaped Helmholtz-capacitance response and with the reduced capacitance at larger positive and negative charging. A detailed analysis of this nonlinearity, and of the asymmetry between positive and negative bias, is beyond the scope of the present work.[25,27,35]

In the main text, we focus on the central charging regime near the PZC, corresponding to the unshaded region in Fig. 1. This regime spans approximately -10 to 20 $\mu\mathrm{C\,cm^{-2}}$ and is the range used for the capacitance decomposition in Fig. 2 of the main text. The inverse bias capacitances extracted from this regime are compared in Fig. S10. Both the full-cell bias capacitance, C_{bias}, and the interfacial bias capacitance, $C_{\text{bias-interface}}$, show the same metal-dependent trend (Figs. S10a,b) and are linearly correlated (Fig. S10c). This shows that restricting the analysis to the simulated interfacial region preserves the essential metal dependence of the bias response. The interfacial quantity $C_{\text{bias-interface}}$ is therefore used in Fig. 2 of the main text.

S3.1.3 Electron Spillover into Water Layers

As discussed in the main text, $\rho_{\text{electrode}}$ denotes the bias-induced electronic response of the explicit metal–water interfaces and is not strictly confined to the metal slab. Part of this

response extends beyond the nominal metal surface into the interfacial water region. We refer to this field-induced spatial extension as electron spillover. This terminology does not imply covalent metal–water bond formation or static charge transfer, but describes the spatial redistribution of electronic density across the interface under electrification. A more detailed physical discussion is provided in Section S2.2 of the Supporting Information of Ref. 9; here, we focus on how this effect enters the present bias-response analysis.

To compare different metals on the same basis, we focus on the central charging regime, $\sigma \approx -10$ to $20\ \mu\mathrm{C\,cm^{-2}}$, where neighboring charge states differ by one excess electron in the simulation cell. We therefore evaluate the finite-difference electrode response,

$$\Delta\rho_{\mathrm{electrode}}(z;\Delta\sigma) = \rho_{\mathrm{electrode}}(\sigma_{i+1}, z) - \rho_{\mathrm{electrode}}(\sigma_i, z),$$

where $\Delta\sigma = \sigma_{i+1} - \sigma_i$. This finite-difference profile removes the trivial dependence on the total number of excess electrons and shows where the incremental electrode-response charge is accommodated along the surface-normal direction.

For clarity, we denote the finite-difference profile as $\rho_{\mathrm{electrode}}(z)$ in the following discussion, in Fig. S11, and in the main text. This notation emphasizes that the plotted quantity represents the same electrode-response charge distribution, while the finite-difference operation is used only to compare the incremental response among different metals.

For direct comparison, each finite-difference profile is normalized by its integrated value in the interfacial region up to $z = 5.3$ Å:

$$\tilde{\rho}_{\mathrm{electrode}}(z) = \frac{\rho_{\mathrm{electrode}}(z)A}{\int_{z_{\mathrm{min}}}^{5.3\ \text{Å}} \rho_{\mathrm{electrode}}(z)A\,dz},$$

where A is the surface area and z_{min} denotes the lower bound of the analyzed region. With this normalization, each profile integrates to unity over the chosen region and represents the spatial distribution of the incremental electrode-response charge.

Within this representation, electron spillover is identified by finite weight of the normal-

ized electrode-response charge in the interfacial water region. As shown in Fig. S11, most of the electrode-response charge remains localized near the metal surface, but a finite contribution extends into the interfacial water layer. The extent of this spillover varies across the metal series, indicating that the interfacial solvent participates in the electronic screening response.

The physical implication is that electron spillover changes the effective separation between the electrode-response charge and the compensating countercharge. A more extended electrode response toward the electrolyte reduces the bias-induced potential drop associated with charging. Stronger electron spillover therefore corresponds to stronger interfacial electronic screening and a larger bias-capacitance contribution.

S3.1.4 Model Interfacial Bias Capacitance

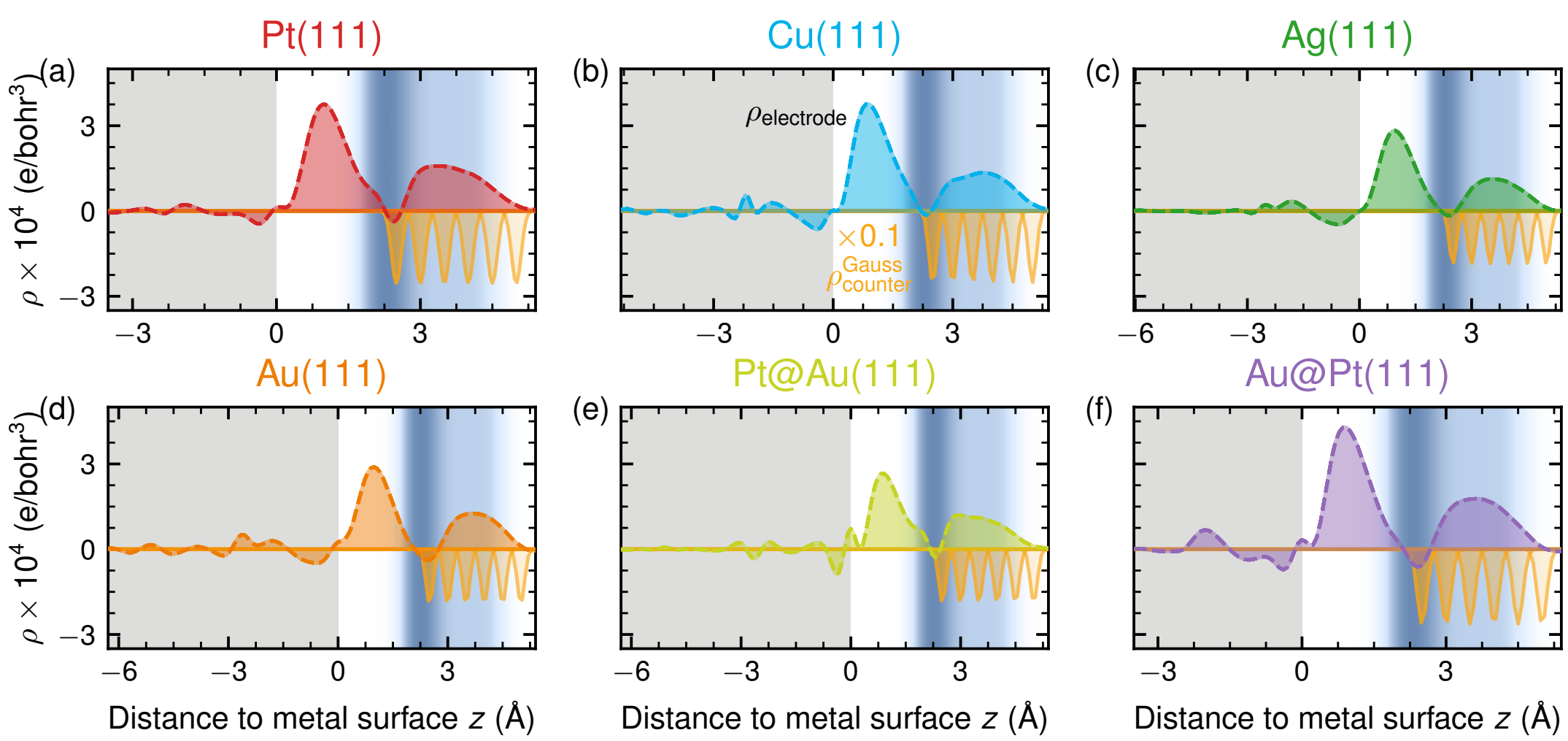

Fig. S12: Interfacial electrode-response and Gaussian countercharge model used in the bias-capacitance analysis. Colored curves show the interfacial electrode response, $\rho_{\mathrm{electrode}}(z)$, after removal of residual bulk-water contributions; orange curves show the Gaussian countercharge model, $\rho^{\mathrm{Gauss}}_{\mathrm{counter}}(z)$. Gray and blue shading indicate the metal slab and interfacial water region, respectively.

To construct a simple electrostatic model for the bias capacitance, we use the interfacial electrode-response profile defined in Section S3.1.3. Residual excess charge in the bulk-water region, $z > 5.3$ Å, is removed, and the remaining interfacial profile. This yields

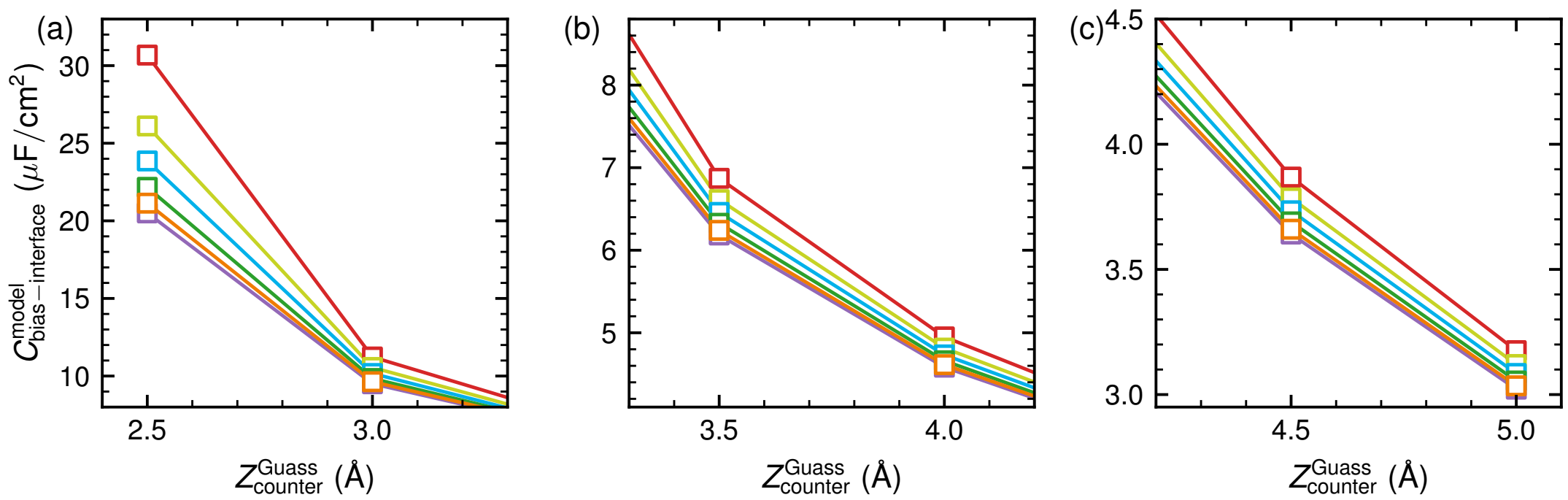

Fig. S13: Model interfacial bias capacitance obtained from the interfacial electrode-response and Gaussian countercharge model shown in Fig. S12. The model capacitance is plotted as a function of the Gaussian countercharge position, $Z^{\text{Gauss}}_{\text{counter}}$. Although the absolute values depend on the assumed countercharge position, the same metal-dependent trend is retained throughout.

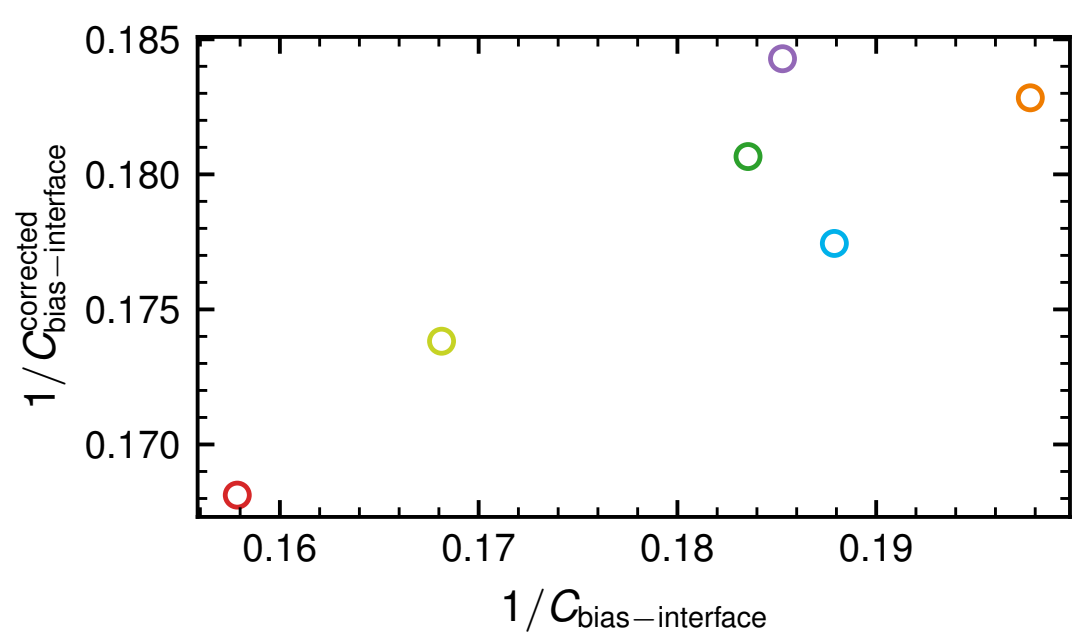

Fig. S14: Comparison between the raw interfacial bias capacitance, $1/C_{\text{bias-interface}}$, and the model interfacial bias capacitance, $1/C^{\text{model}}_{\text{bias-interface}}$.

the interfacial electrode response, $\rho_{\text{electrode}}(z)$, used in the main-text analysis and shown in Fig. S12. The compensating interfacial charge is represented by a Gaussian distribution, $\rho^{\text{Gauss}}_{\text{counter}}(z)$, also shown in Fig. S12. Solving Poisson's equation, Eq. S4, with $\rho_{\text{electrode}}(z)$ and $\rho^{\text{Gauss}}_{\text{counter}}(z)$, and evaluating the resulting capacitance using Eq. S2, yields the model interfacial bias capacitance shown in Fig. S13.

The choice of the Gaussian countercharge is centered at $Z^{\text{Gauss}}_{\text{counter}} = 3.6$ Å in main text. This value was not fitted or optimized to reproduce the DFT-AIMD capacitance trend, but was chosen as a representative near-PZC interfacial countercharge position. Explicit-ion DFT-AIMD simulations of Ag(111)/water show that the equilibrium ClO_4^- distribution exhibits its first interfacial probability maximum at approximately 3.5–3.6 Å from the metal

surface, consistent with experimental structural estimates.[6,10,36,37] As an independent electrostatic consistency check, using the Pt(111) DFT-AIMD $C_{\mathrm{bias-interface}}$ and $Z_{\mathrm{electrode}}$ in the parallel-plate relation introduced in Section S4.1 gives $Z_{\mathrm{counter}} \approx 3.6$ Å. Thus, independent structural and electrostatic estimates support 3.6 Å as a physically reasonable interfacial length scale rather than a fitted parameter.

We emphasize that this position is not universal and can vary with ion identity, concentration, and applied potential. We therefore varied $Z_{\mathrm{counter}}^{\mathrm{Gauss}}$ over a broad interfacial range. As expected for a capacitor-like model, the absolute capacitance changes with the assumed countercharge position. Crucially, however, the metal-dependent trend and its correlation with $Z_{\mathrm{electrode}}$ are retained throughout the representative range considered here (Fig. S13). Thus, the model trend is controlled primarily by the metal-dependent interfacial $\rho_{\mathrm{electrode}}$ profiles rather than by the specific Gaussian countercharge position.

A comparison with the raw interfacial bias capacitance is shown in Fig. S14. The model preserves the overall trend while avoiding over-interpretation of charging-protocol-specific details. In real electrochemical systems, the compensating charge distribution may also depend on electrolyte identity and ion positions in the EDL. Such ion-specific effects are not explicitly captured by the present PCH model and remain an important direction for future work.

S3.1.5 Origin of Ag–Cu Variation in the Interfacial Bias Capacitance

For the whole cell, $1/C_{\mathrm{bias}} = \partial\Delta\phi_{\mathrm{bias}}/\partial\sigma$, where $\partial\sigma = e/A$ corresponds to one excess electron. The corresponding potential response is determined by the first moments of the incremental electrode-response and countercharge distributions,

$$\partial\Delta\phi_{\mathrm{bias}} \propto \int_{-\mathrm{slab}}^{L_z} z\rho_{\mathrm{electrode}}(z)\,dz + \int_{-\mathrm{slab}}^{L_z} z\rho_{\mathrm{counter}}(z)\,dz,$$

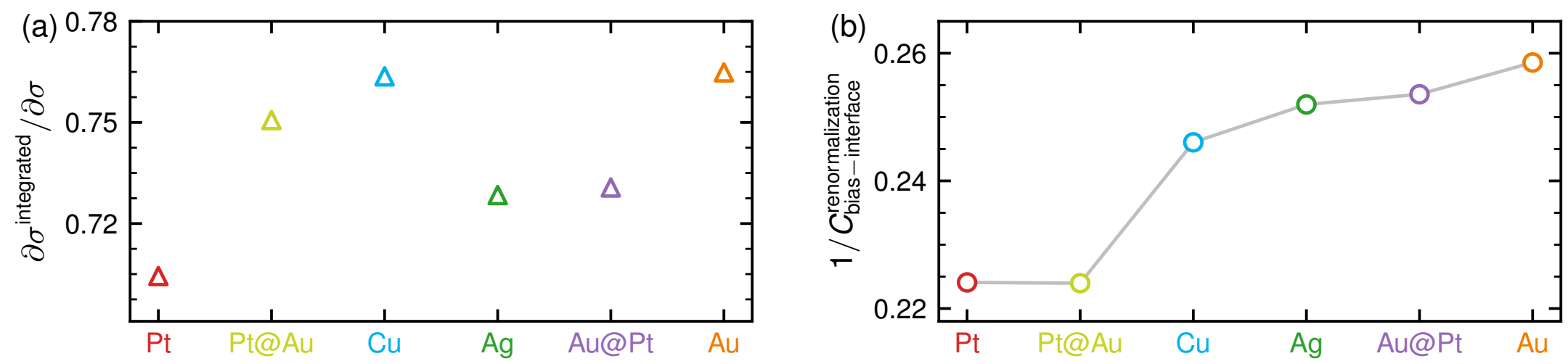

Fig. S15: Metal-dependent partitioning of the excess electronic response. (a) Fraction of the nominal excess charge retained within the interfacial region, $\partial\sigma^{\text{integrated}}/\partial\sigma$. (b) Interfacial-charge-normalized inverse bias contribution, obtained by dividing $1/C_{\text{bias-interface}}$ by $\partial\sigma^{\text{integrated}}/\partial\sigma$. Removing this charge-partitioning factor eliminates the small local Cu–Ag inversion while preserving the overall metal-dependent trend.

with

$$\int_{-\text{slab}}^{L_z} \rho_{\text{electrode}}(z)\,dz = -\int_{-\text{slab}}^{L_z} \rho_{\text{counter}}(z)\,dz = \partial\sigma.$$

Defining the corresponding charge centers as

$$Z_{\text{electrode}} = \frac{\displaystyle\int z\rho_{\text{electrode}}(z)\,dz}{\displaystyle\int \rho_{\text{electrode}}(z)\,dz}, \qquad Z_{\text{counter}} = \frac{\displaystyle\int z\rho_{\text{counter}}(z)\,dz}{\displaystyle\int \rho_{\text{counter}}(z)\,dz},$$

the inverse bias capacitance scales with their effective separation,

$$\frac{1}{C_{\text{bias}}} \propto d, \qquad d = Z_{\text{electrode}} - Z_{\text{counter}}.$$

Since Z_{counter} varies much less than $Z_{\text{electrode}}$ across the metal series, the metal dependence of $1/C_{\text{bias}}$ predominantly tracks $Z_{\text{electrode}}$. For the whole cell, however, $Z_{\text{electrode}}$ also contains the residual response extending into the bulk-water region. Because the partitioning of this response between the interfacial and bulk-water regions is metal dependent (Fig. S15a), it shifts the full-cell $Z_{\text{electrode}}$ by different amounts and can obscure the physically relevant interfacial response.

We therefore restrict the analysis to $z \leq 5.3$ Å, removing the residual bulk-water contribution. The resulting interfacial $Z_{\text{electrode}}$ reflects the spatial distribution of the retained

electrode response and is invariant to its overall magnitude. The fraction of electrode-response charge retained within this region, however, remains metal dependent (Fig. S15a). Consequently, for the metal-dependent variation,

$$\frac{1}{C_{\mathrm{bias-interface}}} \propto \frac{\partial \sigma^{\mathrm{integrated}}}{\partial \sigma}\, d^{\mathrm{IF}} \sim \frac{\partial \sigma^{\mathrm{integrated}}}{\partial \sigma}\, Z^{\mathrm{IF}}_{\mathrm{electrode}},$$

where the second relation follows from the much weaker variation of the countercharge center. Thus, the raw $1/C_{\mathrm{bias-interface}}$ contains both the interfacial charge fraction and the underlying spatial response.

To compare all metals on the same interfacial-charge basis, we divide $1/C_{\mathrm{bias-interface}}$ by $\partial \sigma^{\mathrm{integrated}}/\partial \sigma$. The resulting response retains the overall metal-dependent trend while removing the small local Cu–Ag inversion (Fig. S15b). Thus, restricting the response to the physically relevant interfacial region removes the residual bulk-water contribution, while placing all metals on the same interfacial-charge basis removes differences in the amount of response charge retained within that region. The preservation of the metal-dependent trend on this common charge basis supports $Z^{\mathrm{IF}}_{\mathrm{electrode}}$, which depends only on the spatial interfacial response, as a robust physical descriptor of the metal-dependent electrode response.

This conclusion is further supported by the complementary model introduced in the main text. There, the residual bulk-water response is removed while the interfacial $\rho_{\mathrm{electrode}}$ distributions are retained, and each is combined with an equal-and-opposite Gaussian countercharge having the same spatial form and position across the metal series. The resulting model again reproduces the metal-dependent capacitance trend. Together, the interfacial charge-normalization analysis and the Gaussian model show that the metal-dependent trend is robust to both charge partitioning and countercharge representation and is encoded in the spatial interfacial response characterized by $Z_{\mathrm{electrode}}$.

S3.2 Water-Screening Response

As discussed in the main text and in our previous work,[5] the dominant solvent-side contribution arises from the interfacial-water layer. In the present analysis, the interfacial-water region is defined by a metal-dependent boundary: 4.5 Å for Pt(111), Pt@Au(111), and Cu(111), and 4.8 Å for Ag(111), Au(111), and Au@Pt(111). As discussed in Section S5, these boundaries are determined from the corresponding interfacial water-density profiles in Fig. S22.

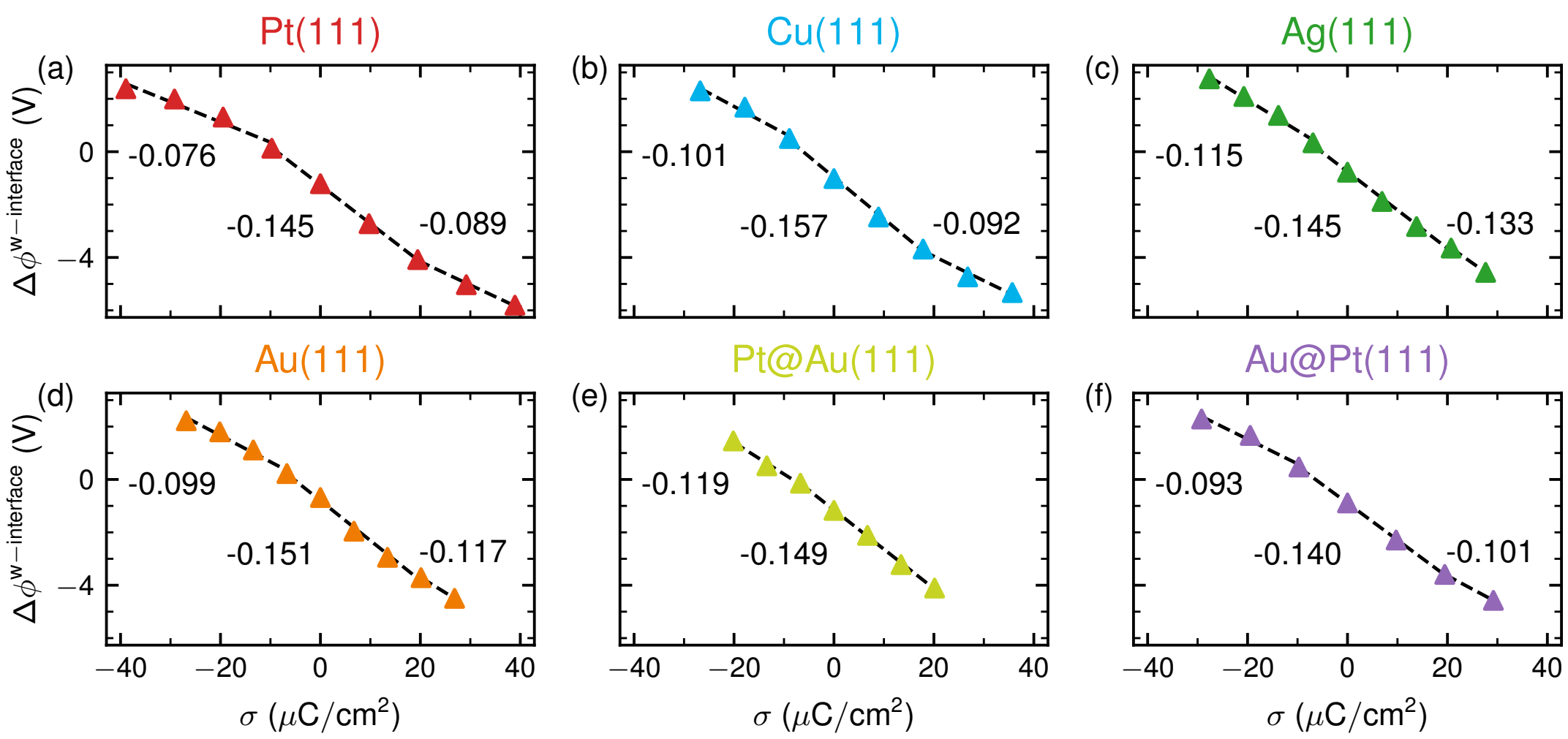

Fig. S16: Surface-charge-dependent potential drops for the interfacial-water contribution, $\Delta\phi^{\mathrm{w-interface}}(\sigma)$, for the different metals. Numbers in each panel indicate the fitted slopes in the different charging regimes, corresponding to the inverse sub-capacitances.

The corresponding surface-charge-dependent potential drops are shown in Fig. S16, and the fitted slopes in the different charging regimes define the corresponding inverse sub-capacitances. The interfacial-water contribution, $\Delta\phi^{\mathrm{w-interface}}(\sigma)$, is obtained by removing the bulk water from the AIMD configurations while retaining the metal slab and the interfacial water layer, and then taking the work-function reduction of this truncated interface relative to that of the clean metal slab. This construction isolates the contribution of interfacial water. The fitted values used in the main text are taken from the peak regime, approximately -10 to 20 $\mu\mathrm{C\,cm^{-2}}$, and are summarized in Fig. 2 of the main text.

S4 Electrostatic Significance and Robustness of $Z_{\text{electrode}}$

S4.1 Electrostatic Significance

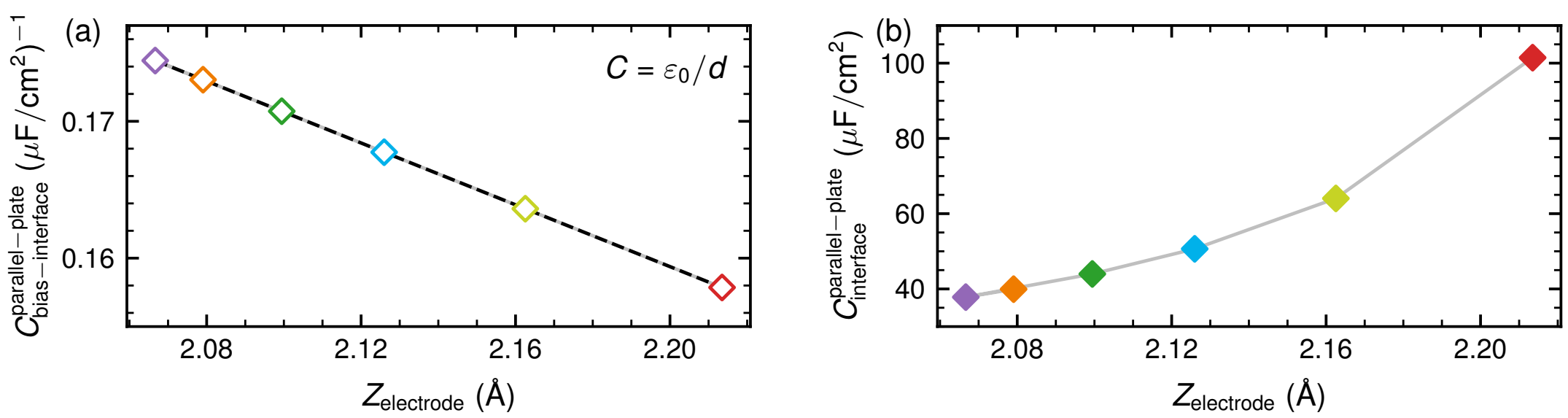

Fig. S17: Electrostatic sensitivity of the interfacial capacitance to $Z_{\text{electrode}}$. (a) Parallel-plate estimate of the inverse interfacial bias contribution, $1/C^{\text{PP}}_{\text{bias-interface}} = d/\varepsilon_0$, with $d = |Z_{\text{electrode}} - Z_{\text{counter}}|$ and a common Z_{counter} for all metals. (b) Corresponding interfacial capacitance obtained by additionally including the average interfacial-water contribution (see Fig. 2c). The analysis illustrates how the ~ 0.1 Å metal-dependent variation in $Z_{\text{electrode}}$ translates into a substantial variation in the capacitance.

Although the metal-dependent variation in $Z_{\text{electrode}}$ is only ~ 0.1 Å in absolute terms, the interfacial potential drop is established over a very short electrostatic length scale, corresponding to a strong interfacial field. On this scale, even a sub-Å displacement of the electrode-response charge center can appreciably alter the potential drop and capacitance. To quantify how this variation affects the interfacial capacitance, we first estimate the interfacial bias contribution using a simple parallel-plate model,

$$\frac{1}{C^{\text{PP}}_{\text{bias-interface}}} = \frac{d}{\varepsilon_0}, \qquad d = |Z_{\text{electrode}} - Z_{\text{counter}}|\,.$$

A common Z_{counter} is used for all metals. Its position is determined from the Pt(111) DFT-AIMD values of $C_{\text{bias-interface}}$ and $Z_{\text{electrode}}$, and is then kept fixed for all other metals. The metal dependence of $C^{\text{PP}}_{\text{bias-interface}}$ therefore arises directly from the calculated variation in $Z_{\text{electrode}}$. Only the vacuum permittivity, $\varepsilon_0 = 8.854 \times 10^{-12}$ F m^{-1}, enters this bias-only estimate because the water response is treated explicitly as a separate contribution in our

decomposition rather than through an effective dielectric constant. As shown in Fig. S17a, the ~ 0.1 Å variation in $Z_{\mathrm{electrode}}$ produces a clear variation in the estimated bias contribution.

To assess how this variation propagates to the interfacial capacitance, we combine the estimated bias contribution with the average interfacial-water contribution from Fig. 2c of the main text,

$$\frac{1}{C^{\mathrm{PP}}_{\mathrm{interface}}} = \frac{1}{C^{\mathrm{PP}}_{\mathrm{bias-interface}}} + \frac{1}{C_{\mathrm{w-interface}}},$$

where $1/C_{\mathrm{w-interface}} = -0.148\ (\mu\mathrm{F\,cm^{-2}})^{-1}$. The resulting $C^{\mathrm{PP}}_{\mathrm{interface}}$ exhibits a substantial metal-dependent variation (Fig. S17b). Thus, although the ~ 0.1 Å variation in $Z_{\mathrm{electrode}}$ is small in absolute structural terms, it is significant on the short electrostatic length scale governing the Helmholtz response.

This analysis shows that the ~ 0.1 Å metal-dependent variation in $Z_{\mathrm{electrode}}$ is electrostatically significant. We next assess whether this variation is sufficiently resolved relative to numerical and finite-sampling uncertainties.

S4.2 Robustness with Respect to the Spatial Boundary

The metal-dependent bias response is established independently of the spatial truncation used to define $Z_{\mathrm{electrode}}$. As shown in Fig. S10, the full-cell and interfacial inverse bias capacitances, $1/C_{\mathrm{bias}}$ and $1/C_{\mathrm{bias-interface}}$, exhibit the same metal-dependent trend ($R^2 = 0.996$). The spatial boundary is therefore introduced only to construct a compact descriptor of the interfacial electronic response.

The reference boundary at $z = 5.3$ Å is motivated by the spatially resolved response profiles in Fig. S18. For all six systems, it encompasses the characteristic second spillover feature within the first interfacial water layer while excluding the residual response extending further into bulk water.

To assess sensitivity to this interface–bulk partition, we reevaluated $Z_{\mathrm{electrode}}$ using common boundaries of $z = 4.6$, 5.3, and 6.0 Å. The 4.6 Å boundary still retains the characteristic

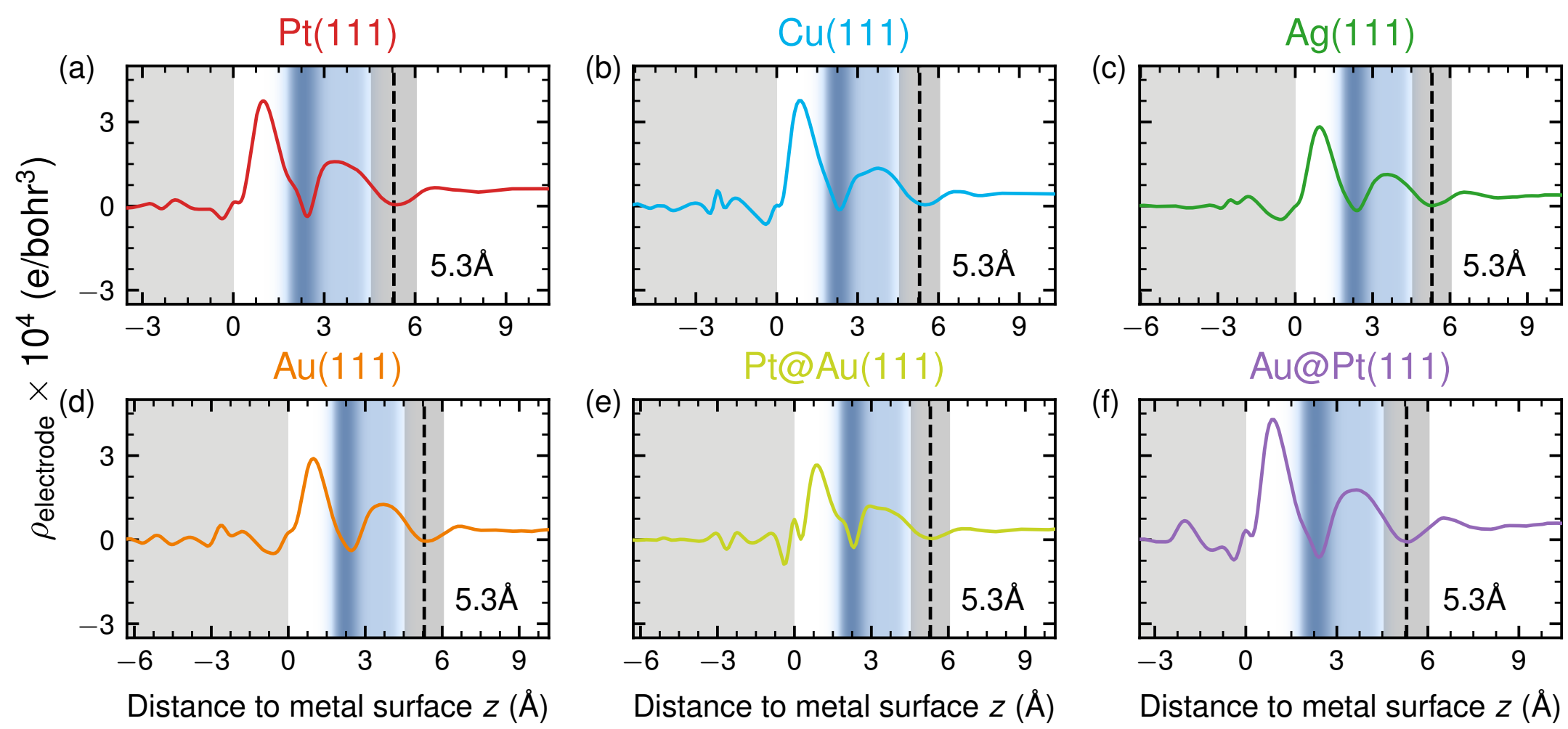

Fig. S18: Spatial basis for the reference boundary used to define $Z_{\mathrm{electrode}}$. Bias-induced electronic-response profiles, $\rho_{\mathrm{electrode}}$, for the six metal systems, derived from Fig. S8. The reference boundary at z = 5.3 Å encompasses the characteristic second spillover feature within the first interfacial water layer while excluding the residual bulk-water response. The black shaded region marks the boundary range z = 4.6–6.0 Å used for the sensitivity analysis in Fig. S19.

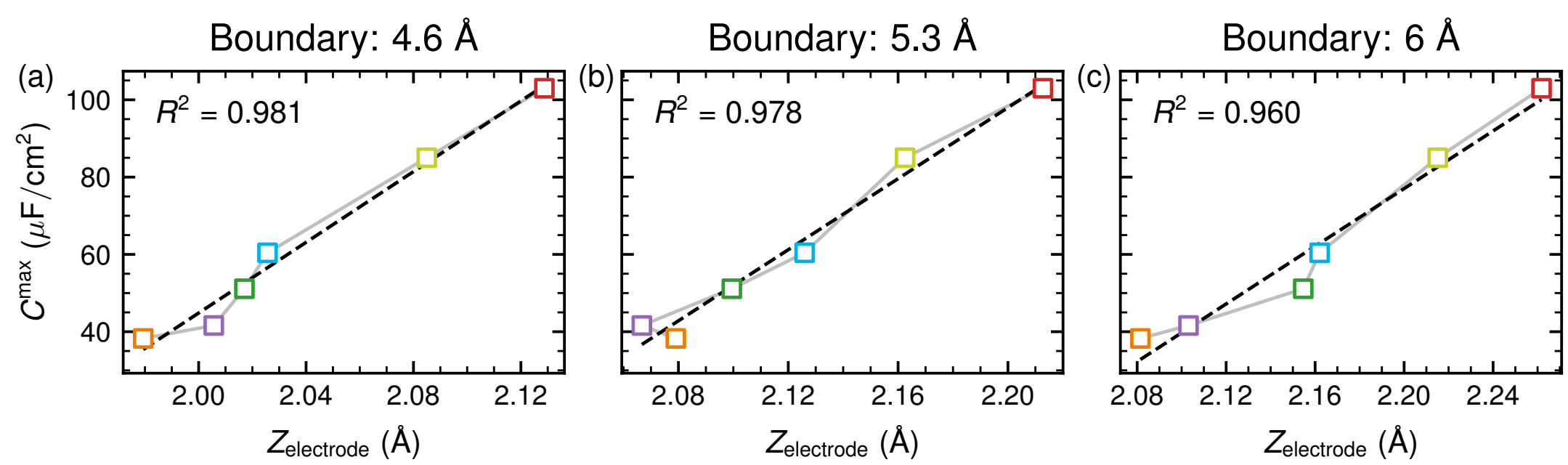

Fig. S19: Robustness of the capacitance–$Z_{\mathrm{electrode}}$ relation to the spatial boundary. Correlation between the DFT-AIMD peak capacitance, C^{max}, and $Z_{\mathrm{electrode}}$ for (a) z = 4.6 Å, (b) z = 5.3 Å, and (c) z = 6.0 Å, corresponding to the boundary range highlighted by the black shaded region in Fig. S18. The restrictive boundary retains the characteristic interfacial spillover feature, whereas the extended boundary includes additional residual bulk-water response. Despite shifts in the absolute $Z_{\mathrm{electrode}}$ values, the strong capacitance correlation is retained (R^2 = 0.981, 0.978, and 0.960, respectively).

interfacial spillover feature, whereas the 6.0 Å boundary includes additional residual bulk-water response. Although the absolute $Z_{\mathrm{electrode}}$ values shift, the strong correlation with the DFT-AIMD peak capacitance is retained, with R^2 = 0.981, 0.978, and 0.960, respectively (Fig. S19). Thus, the metal-dependent capacitance–$Z_{\mathrm{electrode}}$ relation is robust to reasonable

variations of the interface–bulk partition.

S4.3 Numerical Robustness

Table S3: Statistical uncertainty of $Z_{\text{electrode}}$ from block averaging of the AIMD trajectories. Each trajectory was divided into five contiguous time blocks.

System	Pt	Pt (1200eV)	Pt@Au	Cu	Ag	Au	Au@Pt
$Z_{\text{electrode}}$ (Å)	2.21349	2.21383	2.16253	2.12597	2.09945	2.07906	2.06667
SD (Å)	0.02112	0.02039	0.01459	0.01174	0.01039	0.00935	0.02263
SEM (Å)	0.00945	0.00912	0.00653	0.00525	0.00465	0.00418	0.01012

Because our physical interpretation relies on the relative metal-dependent trend in $Z_{\text{electrode}}$ rather than its absolute value, we assess whether the observed ~ 0.1 Å variation is well resolved and robust against the plane-wave cutoff, simulation-cell dimensions, slab thickness, and finite AIMD sampling.

Plane-wave cutoff. Increasing the plane-wave cutoff from 400 to 1200 eV for representative Pt(111) snapshots changes $Z_{\text{electrode}}$ from 2.21349 to 2.21383 Å, i.e., by only 3.4×10^{-4} Å. This difference is negligible compared with the overall metal-dependent variation and the finite-AIMD sampling uncertainty (Fig. S21 and Table S3).

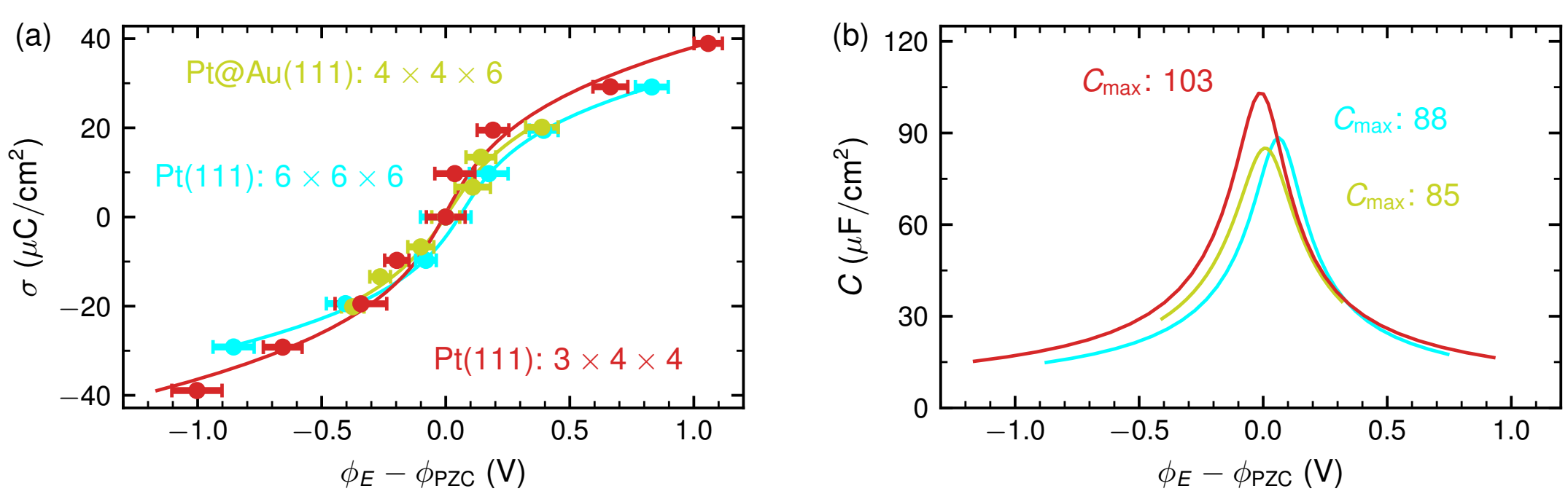

Fig. S20: Sensitivity of the Pt(111)/water capacitance to the lateral cell dimensions and slab thickness. (a) Surface charge density as a function of electrode potential for the production Pt(111) model and enlarged simulation cells. (b) Corresponding differential-capacitance curves. The characteristic peak-shaped capacitance response is retained upon increasing the simulation-cell dimensions.

Cell size and slab thickness. Enlarged Pt(111)/water models were used to assess sensitivity to the lateral cell dimensions and slab thickness. The characteristic surface-charge–potential relation and peak-shaped differential-capacitance response are retained upon increasing the simulation-cell dimensions (Fig. S20). Although the absolute peak capacitance changes moderately, the enlarged Pt(111) $6 \times 6 \times 6$ model remains consistent with the characteristic capacitance–$Z_{\text{electrode}}$ relation (Fig. S21).

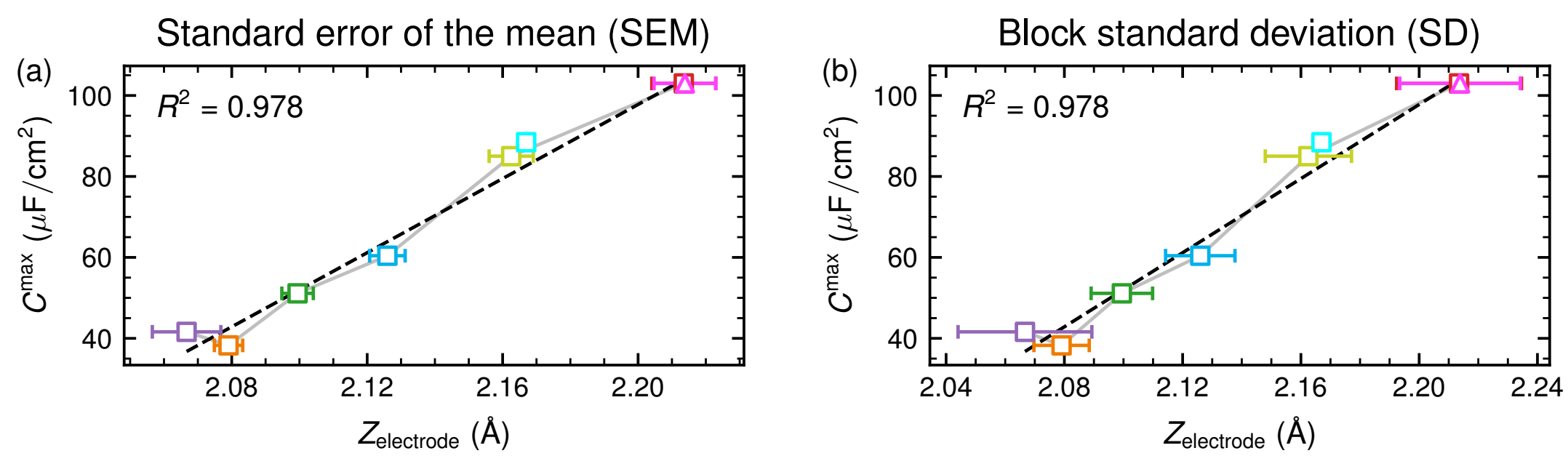

Fig. S21: Statistical uncertainty and numerical robustness of $Z_{\text{electrode}}$. Each AIMD trajectory was divided into five contiguous time blocks, and $Z_{\text{electrode}}$ was evaluated independently for each block. (a) Horizontal error bars denote the standard error of the mean (SEM) of the five block values. (b) Horizontal error bars denote the standard deviation (SD) among the five block values. The additional light-pink Pt(1200 eV) and cyan enlarged Pt(111) $6 \times 6 \times 6$ points denote the plane-wave-cutoff and simulation-cell robustness tests, respectively.

Finite AIMD sampling. Each AIMD trajectory was divided into five contiguous time blocks, and $Z_{\text{electrode}}$ was evaluated independently for each block. The resulting standard deviations are approximately 0.009–0.023 Å, and the corresponding standard errors of the mean are approximately 0.004–0.010 Å (Fig. S21 and Table S3). Both are substantially smaller than the overall ~ 0.1 Å metal-dependent variation, demonstrating that the trend is well resolved relative to finite-trajectory sampling uncertainty.

Together, these analyses show that the metal-dependent variation in $Z_{\text{electrode}}$ is electrostatically significant, statistically well resolved, and robust to representative changes in the plane-wave cutoff, lateral cell size, and slab thickness.

S5 Structural Properties at the Electrified Metal(111)–Water Interface

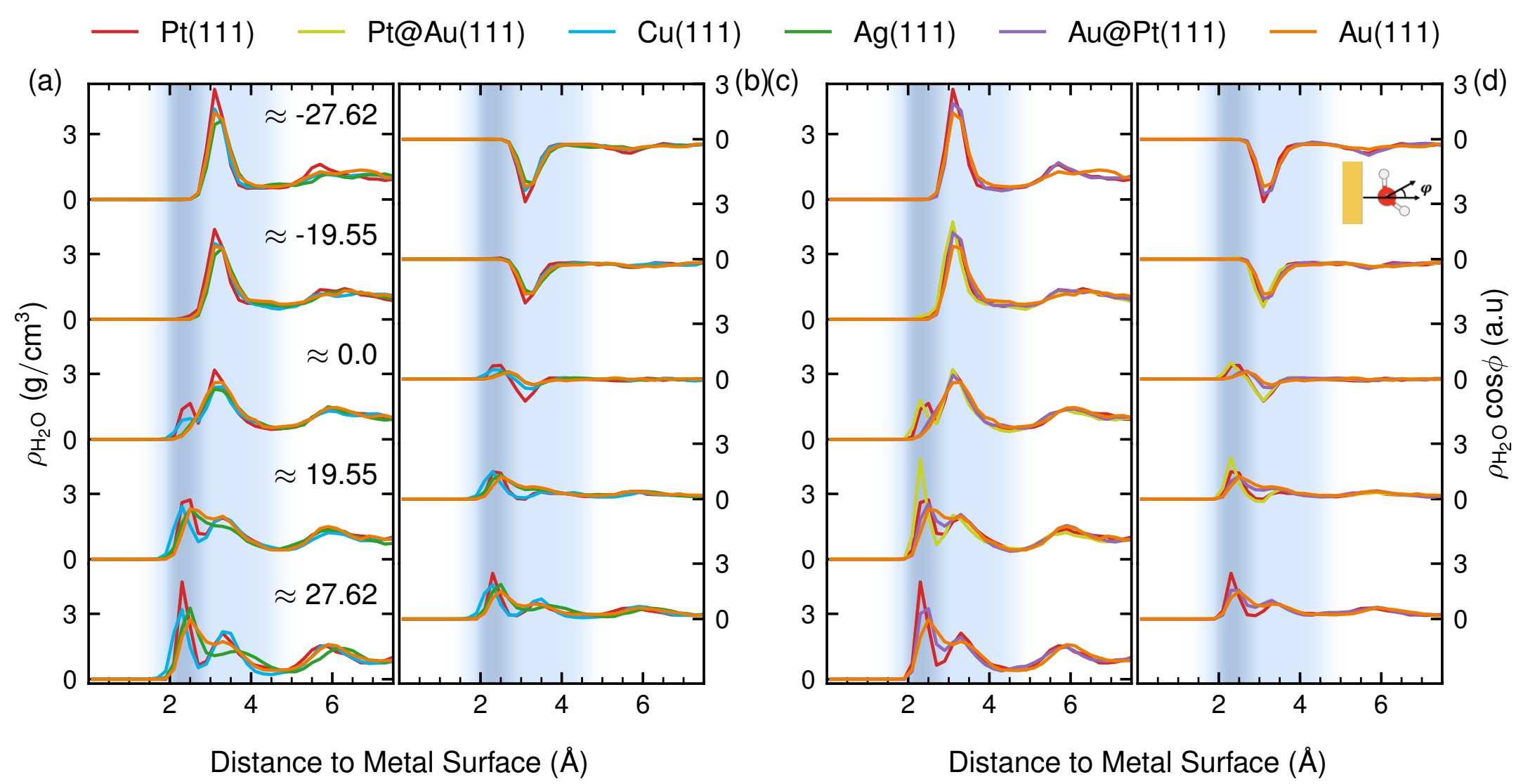

Fig. S22: Interfacial water structure at approximately matched surface charge densities. Panels (a, b) compare Pt(111), Cu(111), Ag(111), and Au(111), whereas panels (c, d) compare Pt(111), Pt@Au(111), Au(111), and Au@Pt(111). Panels (a, c) show the water density distribution, ρ_{H_2O} (g,cm^{-3}), and panels (b, d) show the orientational distribution, $\rho_{H_2O} \cos\varphi$ (a.u.), as functions of distance from the metal surface. From top to bottom, the profiles correspond to $\sigma \approx -27.62$, -19.55, 0.0, 19.55, and 27.62 μC,cm^{-2}. The shaded region indicates the approximate interfacial-water region used in the analysis. In panel (d), φ denotes the angle between the water dipole moment and the surface normal.

To compare the interfacial water structure across the metal series on an equal footing, Fig. S22 shows the water density and orientational distributions at approximately matched surface charge densities for Pt(111), Pt@Au(111), Cu(111), Ag(111), Au@Pt(111), and Au(111), namely $\sigma \approx -27.62$, -19.55, 0.0, 19.55, and 27.62 $\mu\mathrm{C\,cm^{-2}}$.

All interfaces exhibit the characteristic bilayer-like structure of interfacial water, with a first peak associated with the interfacial layer and a broader second feature at larger distance from the surface. Already at $\sigma \approx 0$, the density profiles show clear metal dependence, particularly in the position, height, and sharpness of the first-layer peak. Pt(111), Pt@Au(111), and

Cu(111) display a sharper and more pronounced first peak, whereas Ag(111), Au@Pt(111), and Au(111) exhibit broader first-layer features and a less structured second layer. These differences indicate systematic variations in metal–water interaction strength and interfacial layering across the metal series.

With increasing positive surface charge, the first-layer density peak sharpens and becomes more localized for all metals, while the corresponding orientational profiles develop increasingly positive first-layer features, consistent with stronger preferred alignment of interfacial water dipoles. Upon negative charging, by contrast, the first-layer density profiles broaden and the orientational response shifts in the opposite direction, indicating reversal of the preferred dipolar alignment. Despite these common trends, the magnitude of the structural response remains metal dependent. In particular, Pt(111) and Pt@Au(111) retain stronger first-layer structuring across the full charging range, whereas Au(111) and Ag(111) exhibit a weaker and more diffuse interfacial response.

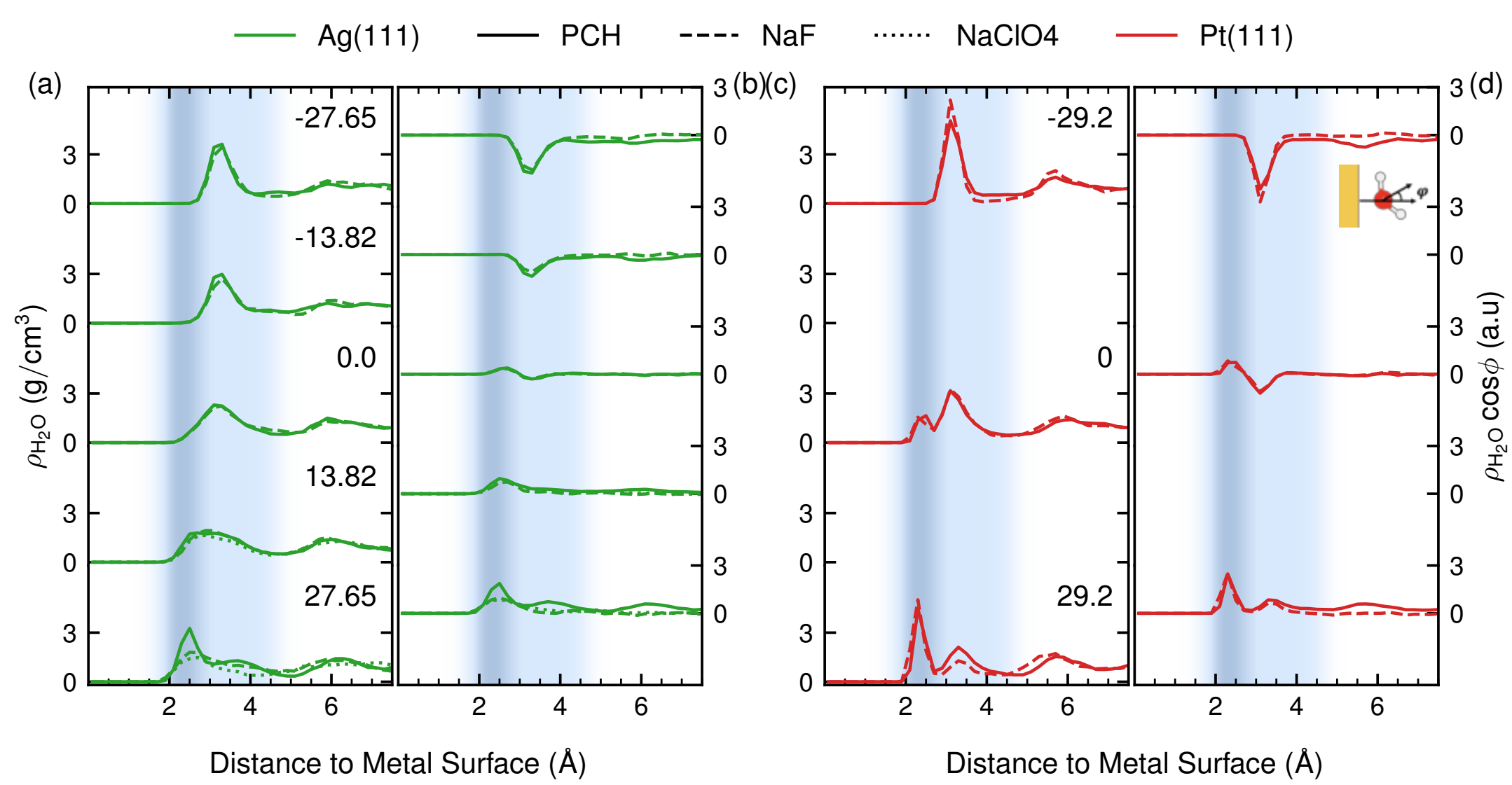

Fig. S23: Comparison of interfacial-water responses from PCH and explicit-ion DFT-AIMD for (a,b) Ag(111) and (c,d) Pt(111). The main potential-dependent changes in water density and orientation are qualitatively retained across the different countercharge representations. Pt(111) PCH data reproduced from Ref. 5. Copyright 2025 American Chemical Society; Ag(111) explicit-ion data adapted with permission from Ref. 6. Copyright 2022 Elsevier; Pt(111) explicit-ion data from Ref. 10. For Ag(111), this agreement is particularly evident over the peak regime (~ -10 to 20 μC cm^{-2}).

The comparison at matched surface charge density shows that σ provides a useful descriptor for comparing water-structure changes across different metals. At the same time, the systematic differences between the metals show that the interfacial water response is not universal, but instead reflects the underlying metal-dependent interaction with water.

The robustness of the interfacial-water response with respect to the countercharge representation is further supported by explicit-ion DFT-AIMD benchmarks (Fig. S23). For Pt(111), our previous comparison showed only marginal differences in water density and orientation between PCH and explicit-counterion simulations.[5,10] Explicit-ion simulations of Ag(111) likewise show comparable potential-dependent water reorganization, particularly over the peak regime (~ -10 to 20 $\mu\mathrm{C\,cm^{-2}}$).[6] Thus, while ion-specific interactions may quantitatively modify the interfacial structure, the principal water response captured by the PCH approach is qualitatively retained.

S6 Bare-Metal Descriptors Fail to Capture Interfacial Spillover

The main text shows that the electron-spillover descriptor, $Z_{\mathrm{electrode}}$, captures the metal-dependent trend in the Helmholtz capacitance. Since $Z_{\mathrm{electrode}}$ describes the spatial extent of the interfacial electrode response, a natural question is whether this descriptor can be inferred from intrinsic properties of the clean metal surface. To test this possibility, we evaluated several electronic descriptors for the corresponding metal(111) slabs in vacuum, including the work function, Φ_M, the occupied d-band center, $\varepsilon_d - E_F$, the total density of states near the Fermi level, $\langle \mathrm{DOS} \rangle_{\mathrm{near}\ E_F}$, and the d-projected density of states.

As shown in Fig. S25, neither the work function nor the occupied d-band center follows the trend in $Z_{\mathrm{electrode}}$. Similarly, the total DOS profiles and the DOS near E_F do not reproduce the $Z_{\mathrm{electrode}}$ trend (S27). Although the d-projected DOS differs clearly among the bare metal surfaces, it also does not provide a direct descriptor for $Z_{\mathrm{electrode}}$ (Fig. S24).

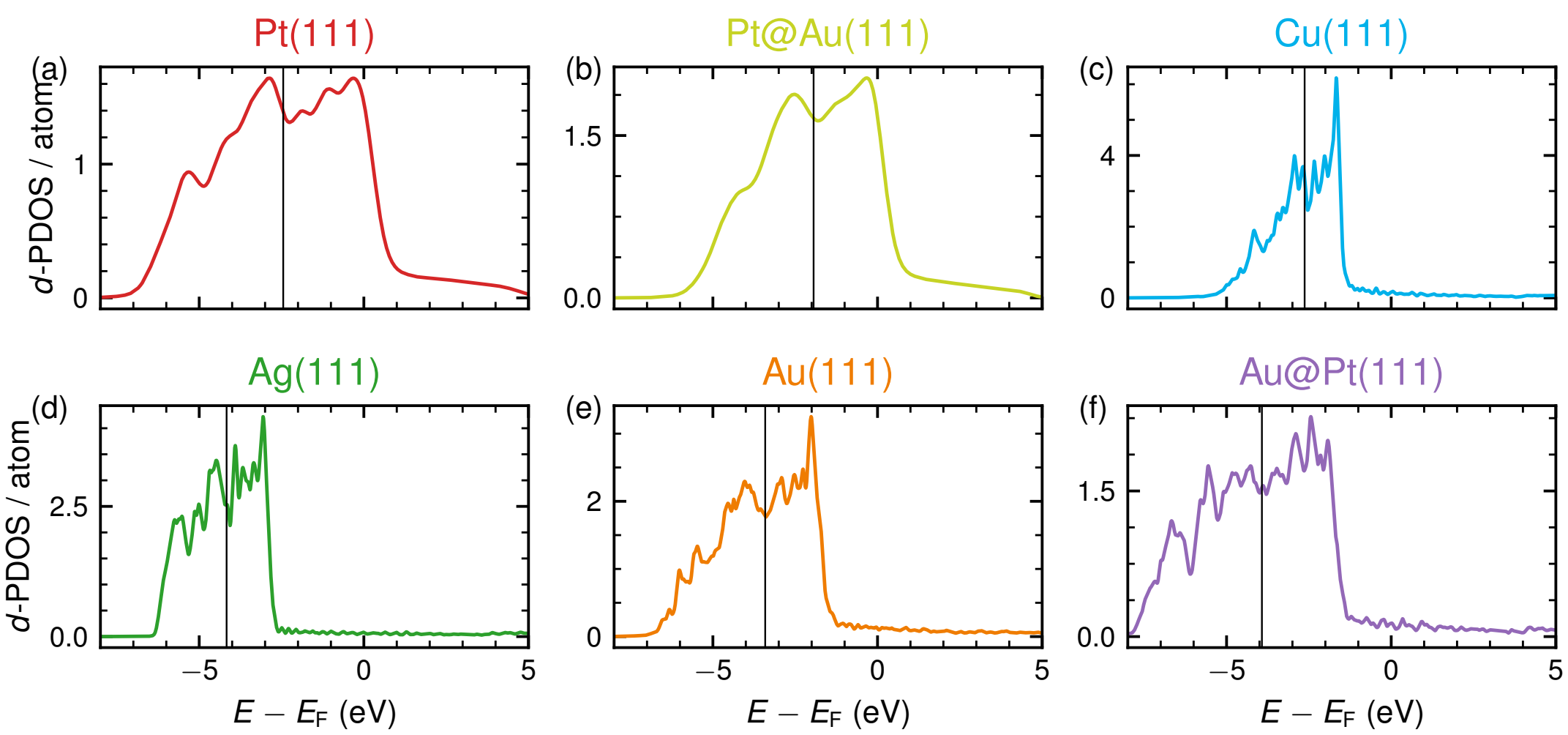

Fig. S24: d-projected density of states (d-PDOS) for Pt(111), Pt@Au(111), Cu(111), Ag(111), Au(111), and Au@Pt(111), plotted relative to the Fermi level. The vertical black lines indicate the occupied d-band center, $\varepsilon_d - E_F$.

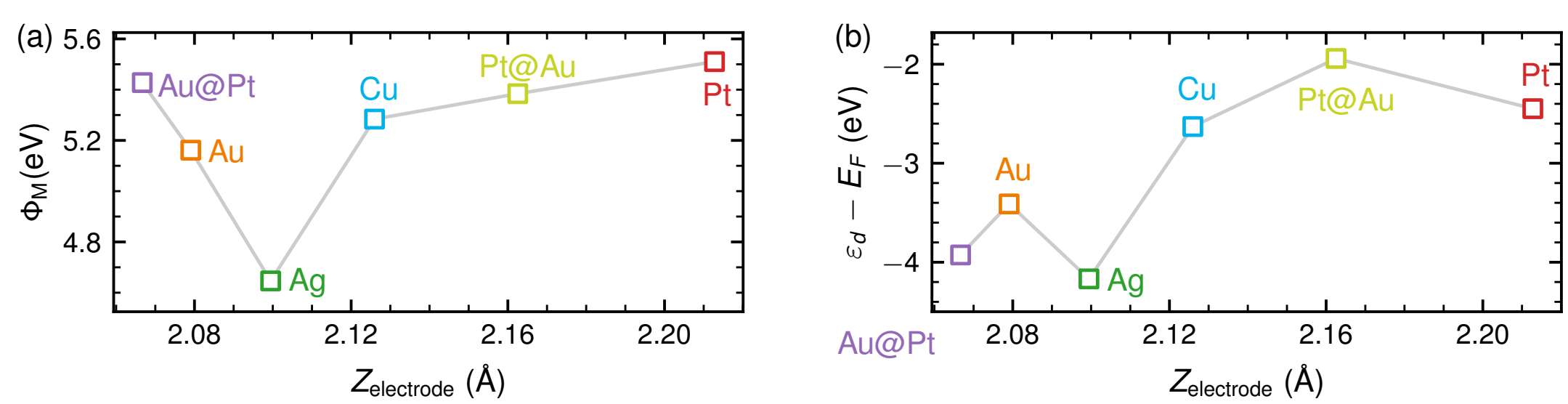

Fig. S25: (a) Work function, Φ_M, and (b) occupied d-band center, $\varepsilon_d - E_F$, obtained from Fig. S24, as functions of the electrode position, $Z_{\text{electrode}}$, for Pt(111), Pt@Au(111), Cu(111), Ag(111), Au(111), and Au@Pt(111).

These results indicate that $Z_{\text{electrode}}$ is not determined solely by the intrinsic electronic structure of the isolated metal surface. Instead, it reflects an interfacial electronic response that emerges at the explicitly charged metal–water interface. This supports the conclusion of the main text that the metal-dependent Helmholtz capacitance is governed by electron spillover modified by interfacial water, rather than by bare-metal descriptors alone.

The DOS and PDOS analyses in this section were performed using the Vienna Ab initio Simulation Package (VASP)[38–40] with projector-augmented wave (PAW) pseudopotentials.[41] All computational parameters were kept consistent with those used in the CP2K calculations described in Section S1.2. The supercell details for each system are provided in Table S1. To

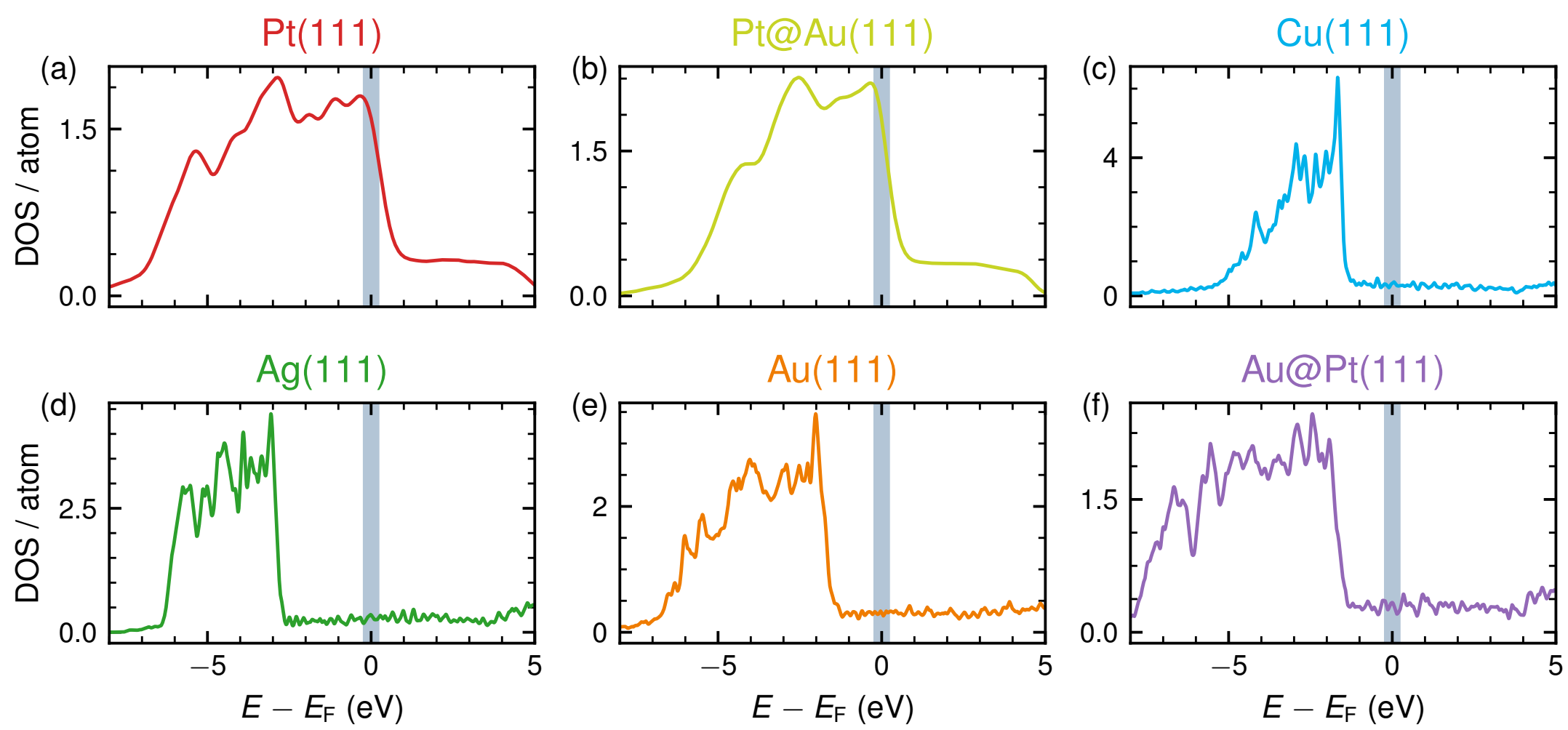

Fig. S26: Total density of states (DOS) per atom for Pt(111), Pt@Au(111), Cu(111), Ag(111), Au(111), and Au@Pt(111), plotted relative to the Fermi level. The vertical gray shaded region indicates the energy window used to evaluate the DOS near E_F.

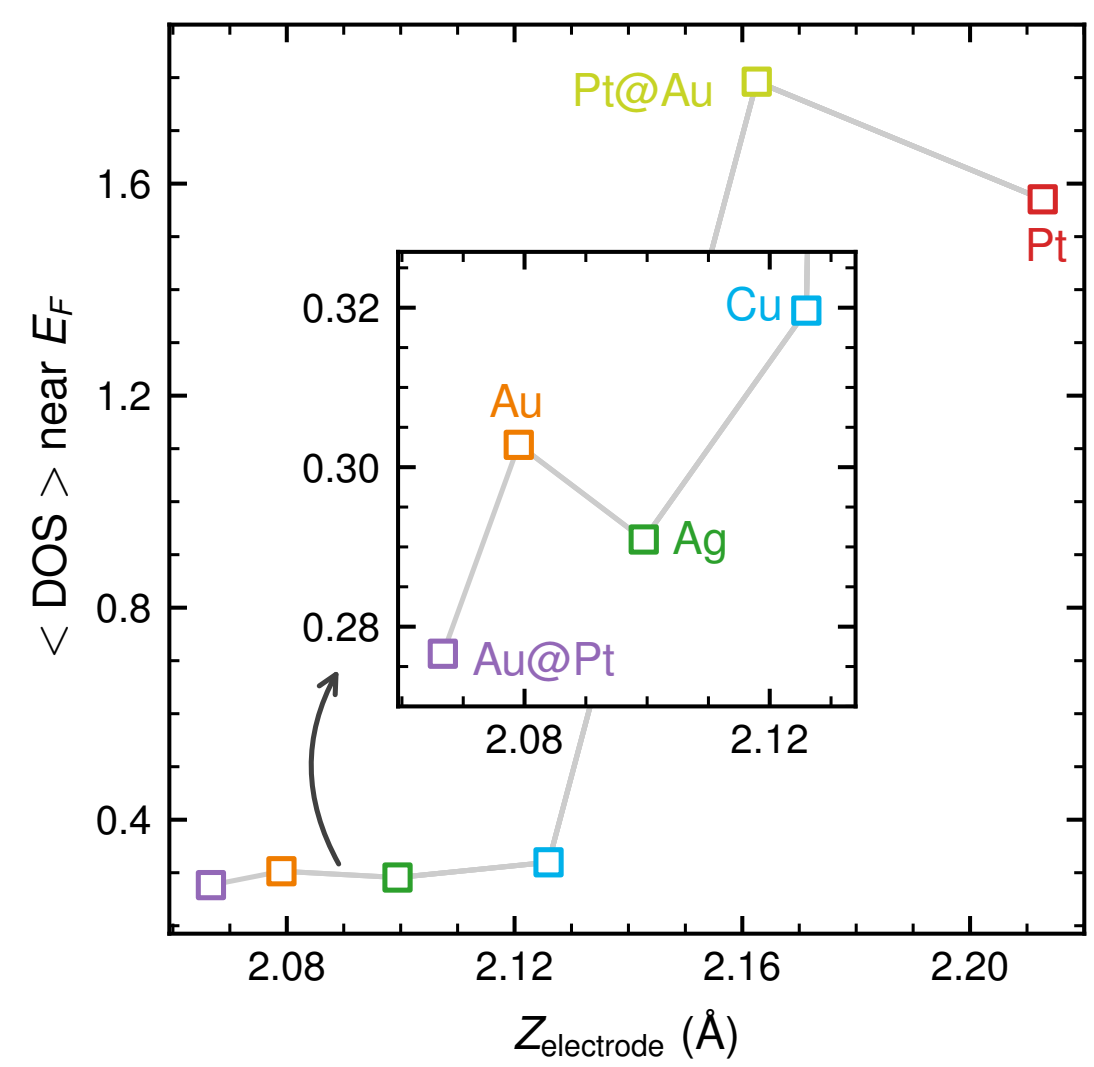

Fig. S27: DOS near the Fermi level, $\langle \text{DOS} \rangle_{\text{near } E_F}$, extracted from the gray-shaded energy window in Fig. S26, as a function of the electrode position, $Z_{\text{electrode}}$. The inset shows a zoomed-in view of the low-DOS region.

reduce the computational cost associated with dense k-point sampling, a two-step procedure was used. First, a self-consistent calculation was performed using a centered $3 \times 2 \times 1$ k-point grid for Pt and a $2 \times 2 \times 1$ grid for the other systems to obtain the converged charge density. Then, starting from this charge density, a non-self-consistent calculation was performed using

a denser $11 \times 8 \times 1$ k-point grid for Pt and an $8 \times 8 \times 1$ grid for the other systems to obtain the DOS and PDOS.

References

(1) Michaelson, H. B. The work function of the elements and its periodicity. *J. Appl. Phys.* **1977**, *48*, 4729–4733.

(2) Trasatti, S.; Lust, E. In *Modern Aspects of Electrochemistry No. 33*; White, R. E., Ed.; Kluwer Academic Publishers, 2002; pp 1–215.

(3) Derry, G. N.; Kern, M. E.; Worth, E. H. Recommended values of clean metal surface work functions. *J. Vac. Sci. Technol. A* **2015**, *33*.

(4) Hörmann, N. G.; Andreussi, O.; Marzari, N. Grand canonical simulations of electrochemical interfaces in implicit solvation models. *J. Chem. Phys.* **2019**, *150*, 041730.

(5) Li, L.; Reuter, K.; Hörmann, N. G. Deciphering the Capacitance of the Pt(111)/Water Interface: A Micro- to Mesoscopic Investigation by AIMD and Implicit Solvation. *ACS Electrochem.* **2025**, *1*, 186–194.

(6) Li, L.; Liu, Y.; Le, J.; Cheng, J. Unraveling molecular structures and ion effects of electric double layers at metal water interfaces. *Cell Rep. Phys. Sci.* **2022**, *0*, 100759.

(7) Le, J.; Iannuzzi, M.; Cuesta, A.; Cheng, J. Determining Potentials of Zero Charge of Metal Electrodes versus the Standard Hydrogen Electrode from Density-Functional-Theory-Based Molecular Dynamics. *Phys. Rev. Lett.* **2017**, *119*, 16801.

(8) Schnur, S.; Groß, A. Properties of metal-water interfaces studied from first principles. *New J. Phys.* **2009**, *11*, 125003.

(9) Li, L.; Eggert, T.; Reuter, K.; Hörmann, N. G. Electron Spillover into Water Layers: A Quantum Leap in Understanding Capacitance Behavior. *J. Am. Chem. Soc.* **2025**, *147*, 22778–22784.

(10) Le, J.; Fan, Q.; Li, J.; Cheng, J. Molecular origin of negative component of Helmholtz capacitance at electrified Pt(111)/water interface. *Sci. Adv.* **2020**, *6*, eabb1219.

(11) Bouzid, A.; Pasquarello, A. Atomic-Scale Simulation of Electrochemical Processes at Electrode/Water Interfaces under Referenced Bias Potential. *J. Phys. Chem. Lett.* **2018**, *9*, 1880–1884.

(12) Pajkossy, T.; Kolb, D. M. Double layer capacitance of Pt(111) single crystal electrodes. *Electrochim. Acta.* **2001**, *46*, 3063–3071.

(13) Vandevondele, J.; Krack, M.; Mohamed, F.; Parrinello, M.; Chassaing, T.; Hutter, J. Quickstep: Fast and accurate density functional calculations using a mixed Gaussian and plane waves approach. *Comput. Phys. Commun.* **2005**, *167*, 103–128.

(14) Hartwigsen, C.; Goedecker, S.; Hutter, J. Relativistic separable dual-space Gaussian pseudopotentials from H to Rn C. *Phys. Rev. B.* **1998**, *58*, 114105.

(15) Goedecker, S.; Teter, M. Separable dual-space Gaussian pseudopotentials. *Phys. Rev. B.* **1996**, *54*, 1703–1710.

(16) VandeVondele, J.; Hutter, J. Gaussian basis sets for accurate calculations on molecular systems in gas and condensed phases. *J. Chem. Phys.* **2007**, *127*, 114105.

(17) Perdew, J. P.; Burke, K.; Ernzerhof, M. Generalized gradient approximation made simple. *Phys. Rev. Lett.* **1996**, *77*, 3865–3868.

(18) Grimme, S.; Antony, J.; Ehrlich, S.; Krieg, H. A consistent and accurate ab initio parametrization of density functional dispersion correction (DFT-D) for the 94 elements H-Pu. *J. Chem. Phys.* **2010**, *132*, 154104.

(19) Clabaut, P.; Fleurat-Lessard, P.; Michel, C.; Steinmann, S. N. Ten Facets, One Force Field: The GAL19 Force Field for Water-Noble Metal Interfaces. *J. Chem. Theory Comput.* **2020**, *16*, 4565–4578.

(20) Lan, J.; Hutter, J.; Iannuzzi, M. First-Principles Simulations of an Aqueous CO/Pt(111) Interface. *J. Phys. Chem. C* **2018**, *122*, 24068–24076.

(21) Kühne, T. D.; Krack, M.; Mohamed, F. R.; Parrinello, M. Efficient and accurate car-parrinello-like approach to born-oppenheimer molecular dynamics. *Phys. Rev. Lett.* **2007**, *98*, 1–4.

(22) VandeVondele, J.; Hutter, J. An efficient orbital transformation method for electronic structure calculations. *J. Chem. Phys.* **2003**, *118*, 4365–4369.

(23) Berendsen, H. J.; Grigera, J. R.; Straatsma, T. P. The missing term in effective pair potentials. *J. Phys. Chem. A* **1987**, *91*, 6269–6271.

(24) Bellier, J. L. J. P. Potentiels de charge nulle d'electrodes monocristallines de cuivre d'orientation (111) et (100) au contact de solutions aqueuses de perchlorate de potassium. *Electrochim. Acta* **1985**, *30*, 1027–1033.

(25) Valette, G. Double layer on silver single crystal electrodes in contact with electrolytes having anions which are slightly specifically adsorbed. Part III. The (111) face. *J. Electroanal. Chem.* **1989**, *269*, 191–203.

(26) Wang, Z.; Chen, M.; Wu, J.; Ji, X.; Zeng, L.; Peng, J.; Yan, J.; Kornyshev, A. A.; Mao, B.; Feng, G. Constant-Potential Modeling of Electrical Double Layers Accounting for Electron Spillover. *Phys. Rev. Lett.* **2025**, *134*, 046201.

(27) Schmickler, W.; Santos, E. *Interfacial Electrochemistry*; Springer: Berlin, Heidelberg, 2010.

(28) Zhen, E.; Chen, Y.; Huang, J. Double-layer capacitance peaks: Origins, ion dependence, and temperature effects. *J. Chem. Phys.* **2025**, *162*, 144702.

(29) Jovic, B. M. EIS and differential capacitance measurements onto single crystal faces in different solutions Part II : Cu(111) and Cu(100) in 0.1M NaOH. *J. Electroanal. Chem.* **2003**, *541*, 13–21.

(30) Łukomska, A.; Sobkowski, J. Potential of zero charge of monocrystalline copper electrodes in perchlorate solutions. *J. Electroanal. Chem.* **2004**, *567*, 95–102.

(31) Parsons, R.; Zobel, F. G. R. The interphase between mercury and aqueous sodium dihydrogen phosphate. *J. Electroanal. Chem.* **1965**, *9*, 333–348.

(32) Ojha, K.; Arulmozhi, N.; Aranzales, D.; Koper, M. T. M. Double Layer at the Pt(111)–Aqueous Electrolyte Interface: Potential of Zero Charge and Anomalous Gouy–Chapman Screening. *Angew. Chem. Int. Ed.* **2020**, *59*, 711–715.

(33) Chen, W.; Ambrosio, F.; Miceli, G.; Pasquarello, A. Ab initio Electronic Structure of Liquid Water. *Phys. Rev. Lett.* **2016**, *117*, 186401.

(34) Kastlunger, G.; Lindgren, P.; Peterson, A. A. Controlled-potential simulation of elementary electrochemical reactions: proton discharge on metal surfaces. *J. Phys. Chem. C* **2018**, *122*, 12771–12781.

(35) Valetie, G. Double layer on silver single crystal electrodes in contact with electrolytes having anions which are slightly specifically adsorbed. Part II. The (100) face. *J. Electroanal. Chem.* **1982**, *138*, 37–54.

(36) Lucas, C. A.; Thompson, P.; Gründer, Y.; Markovic, N. M. The structure of the electrochemical double layer: Ag(111) in alkaline electrolyte. *Electrochem. commun.* **2011**, *13*, 1205–1208.

(37) Liu, Y.; Kawaguchi, T.; Pierce, M. S.; Komanicky, V.; You, H. Layering and Ordering in Electrochemical Double Layers. *J. Phys. Chem. Lett.* **2018**, *9*, 1265–1271.

(38) Kresse, G.; Hafner, J. Ab initio molecular dynamics for liquid metals. *Phys. Rev. B* **1993**, *47*, 558–561.

(39) Kresse, G.; Furthmüller, J. Efficiency of ab-initio total energy calculations for metals and semiconductors using a plane-wave basis set. *Comput. Mater. Sci.* **1996**, *6*, 15–50.

(40) Kresse, G.; Furthmüller, J. Efficient iterative schemes for ab initio total-energy calculations using a plane-wave basis set. *Phys. Rev. B* **1996**, *54*, 11169–11186.

(41) Kresse, G.; Joubert, D. From ultrasoft pseudopotentials to the projector augmented-wave method. *Phys. Rev. B* **1999**, *59*, 1758–1775.
